# Spatial patterns of plant life-forms along elevation and aridity gradients in temperate deserts of northern Xinjiang, China

**DOI:** 10.1371/journal.pone.0362455

**Published:** 2026-11-13

**Authors:** Cheng Fei, Yi Qiang Dong, Shazhou An

**Affiliations:** 1 Hezhou University, Hezhou, Guangxi Zhuang Autonomous Region, China; 2 College of Grassland XinJiang Agricultural University, Urumqi, Xinjiang Uygur Autonomous Region, China; University of Balochistan, PAKISTAN

## Abstract

Located in the arid hinterland of Central Asia, the mountain temperate deserts in Xinjiang are jointly shaped by elevational geographic constraints and the east-west-oriented drought stress gradient. Nevertheless, the joint coupling relationships between these two gradient factors and community-level vegetation structural attributes across woody and herbaceous plant functional groups remain unclarified quantitatively at present. In this study, we investigated four community structural attributes (plant height, vegetation coverage, population density, and aboveground biomass) across four plant life forms, namely shrubs, semi-shrubs, perennial herbs, and annual herbs, along elevational, latitudinal, and longitudinal gradients in five major desert sub-regions of northern Xinjiang: Altay (n = 159), Tacheng–Yili (n = 164), Bortala and Changji–Urumqi(n = 110) and Eastern Tianshan (n = 94).In total, 527 sampling plots were surveyed during the plant growing season from May to mid-September(Desert field surveys conducted each year from May to September for a total of 3 years); plot numbers were unevenly distributed across subregions according to landscape accessibility and habitat representativeness. No temporal repeated measurements on identical permanent plots were conducted, and all analyses were based on one-off spatial snapshot observations from each plot. Trend-fitting, Single-gradient pattern analysis were applied to examine the correlational relationships between geographic gradients and community structural attributes. The results of this study are as follows: (1) Community structural attributes exhibited pronounced spatial heterogeneity and mostly showed unimodal or parabolic trends along the elevational gradient, with relatively high community performance concentrated in the mid-elevation range of 700–1100 m. This observed elevational unimodal pattern is consistent with the expectation of the mid-domain effect, yet such hump-shaped distributions can also arise from climatic, edaphic or topographic filtering and cannot serve as definitive direct empirical evidence for geometric mid-domain constraints. (2) Community-level vegetation performance tended to decline from the relatively humid western subregions (Tacheng-Yili-Bortala) toward the hyper-arid eastern subregions (Changji-Urumqi, Eastern Tianshan), corresponding to the west-to-east decreasing precipitation gradient driven by westerly circulation. (3) Different plant life-forms displayed distinct correlational adaptive patterns along these combined geographic gradients: shrubs and semi-shrubs maintained relatively stable community dominance under drought and low-temperature conditions; perennial herbs tended to occur in relatively mesic mid-elevation habitats; annual herbs showed high responsiveness to water-related environmental variation. (4) Among surveyed subregions, Eastern Tianshan presented the lowest observed community-attribute values, while the Tacheng-Yili subregion exhibited the highest and most stable community structural status. Collectively, our findings demonstrate correlational associations of elevational constraints and the west-east drought gradient with community structural variation in mountain temperate desert plant assemblages. These observational results provide observational reference for developing region-targeted conservation, grazing management and ecological restoration strategies for Xinjiang’s desert grassland ecosystems.

## Introduction

Desert ecosystems account for over one-third of Earth’s total terrestrial land area, and are characterized by extreme aridity, sparse vegetation cover, fragile community structure, and high sensitivity to climate change and anthropogenic disturbances. As a typical representative of temperate inland deserts in Central Asia, the mountain temperate deserts of Xinjiang provide irreplaceable ecological services, including sand stabilization, water conservation, biodiversity maintenance and livestock forage production. Community structural attributes, including plant height, vegetation coverage, population density and aboveground biomass, are core quantitative indicators that reflect vegetation community structure, plant adaptive strategies, ecosystem functional stability and grassland degradation status. Clarifying the large-scale spatial patterns of these structural attributes and their associated environmental correlates is of great significance for the biodiversity conservation and sustainable management of desert grassland ecosystems.Desert ecosystems serve as critical reservoirs of biodiversity in arid regions worldwide, and the quantitative characteristics of their plant communities directly reflect species survival strategies under environmental stress and the state of ecological equilibrium. Under the harsh conditions of extreme aridity, intense solar radiation, and soil nutrient deficiency, desert plant communities maintain ecosystem stability through highly specialized quantitative parameters, including low canopy cover, sparse stand density, and distinct species composition. These traits are not only evolutionary outcomes of natural selection, but also core indicators for deciphering regional ecological processes.

However, significant discrepancies remain in the scientific understanding of the mechanisms shaping desert plant community quantitative characteristics. On one hand, traditional studies have largely focused on the linear driving effects of single environmental factors (e.g., precipitation or soil moisture) on community structure. On the other hand, with the intensification of climate change and the superimposition of anthropogenic disturbances such as grazing and fencing enclosure, community quantitative characteristics exhibit complex nonlinear responses and time-lag effects, leading to inconsistent findings across different study areas and even among microhabitats within the same region. This fragmented understanding of driving mechanisms results in a general lack of generalizability of ecological restoration models constructed based on quantitative characteristics, hindering their ability to accurately guide desertification control practices.Therefore, systematically synthesizing the spatial patterns, environmental response mechanisms, and dynamic evolution rules of desert plant community quantitative characteristics under anthropogenic disturbances, as well as disentangling the interactions between natural drivers and human activities, has become a critical research gap that urgently needs to be addressed in contemporary ecological research.

The height, cover and density of desert plant communities exhibit a complex heterogeneous spatial distribution driven by environmental gradients, anthropogenic disturbances and plant life forms. Early studies in the eastern Junggar Desert demonstrated that local desert communities have simple structure, prominent dominant species and generally low species diversity indices, forming a quantitative structural pattern dominated by a few dominant species [1]. Subsequent studies across different habitats have further revealed the underlying differentiation mechanisms. In Seriphidium transiliense deserts under grazing stress, community height declines with increasing degradation, while density increases and cover follows a unimodal pattern of initial decrease followed by increase, reflecting a nonlinear response to disturbance [2]. In the lower reaches of the Tarim River, shrub cover shows an S-shaped relationship with groundwater depth, whereas herbaceous cover has no significant correlation with groundwater, indicating that hydrological conditions profoundly shape the spatial heterogeneity of riparian communities [3]. Research in the Fukang desert–oasis ecotone further confirms that soil water content is the primary positive driver of community quantitative characteristics, outweighing the effects of organic matter, total nitrogen and total phosphorus, while total nitrogen acts as a negative driving factor [4].Grazing is the dominant anthropogenic disturbance, and its intensity directly structures the spatial differentiation of community height, cover and abundance. Communities in grazing exclusion areas support more complex structure and significantly higher quantitative metrics than heavily grazed sites. Heavy grazing attenuates the marginal effects of hydrothermal conditions on communities, and changes in community density exhibit a time-lag effect that deviates from the response patterns of height and cover [5–7]. As a widely used restoration measure, fencing generally increases community height, cover and aboveground biomass across all degradation levels, but its effects are highly habitat-specific and indicator-dependent: cover of temperate desert grasslands may decrease after fencing, and restoration efficiency is significantly higher in severely degraded areas than in lightly or moderately degraded zones [8–10].

The quantitative characteristics of Artemisia desert communities on the northern slope of the Tianshan Mountains display a clear longitudinal gradient: height, cover, density and biomass increase from west to east and peak in the middle section. Notably, semi-shrub biomass in the eastern segment is 266.51% higher than in the west, while perennial herbs show a gradual decreasing trend. Elevation, soil organic carbon and mean annual temperature are identified as the main environmental drivers of this gradient pattern [11]. Studies on desert steppes in Gaolan County, Gansu Province, further corroborate the dominant role of temperature: minimum temperature of the coldest month and temperature seasonality directly delimit the distribution ranges of key dominant species such as Reaumuria soongorica and Ajania tenuifolia [12].

From a temporal perspective, fluctuations in hydrothermal conditions shape the quantitative dynamics of desert communities through time-lag and cumulative effects. The overall pattern remains consistent: aboveground standing biomass is positively correlated with cumulative precipitation and negatively correlated with cumulative temperature, but grazing disturbance significantly modulates the marginal effects of these natural drivers [7]. At the microhabitat scale, topographic and moisture conditions in Gobi and sand dune habitats strictly constrain species distribution and abundance. Species such as Cornulaca alaschanica are restricted to low-lying microhabitats in the Gobi–desert transition zone, reinforcing that water availability is the core limiting factor from micro to macro scales [3,13].

In terms of interspecific relationships, desert communities generally show non-significant positive associations or no correlation among species. Most species pairs exhibit no significant positive or negative covariation, and only a few pioneer sand-fixing species form stable commensal pairs. Grazing disturbance can alter interspecific affinity by shifting dominant species status, reflecting the relatively independent survival strategies of species in harsh environments [3,13,14]. Taken together, the spatial distribution and temporal dynamics of desert community quantitative characteristics arise from the synergistic action of soil moisture, temperature, groundwater and grazing. Height, cover and density follow divergent response patterns and evolutionary trajectories across environmental gradients, requiring habitat-specific management strategies.

Desert ecosystems cover nearly 40% of the global terrestrial surface, and vegetation distribution patterns, community assembly mechanisms, temporal stability and invasion responses remain central topics in arid ecology. Recent advances in remote sensing, geophysical exploration and multidimensional statistics have shifted research from descriptive pattern mapping to mechanistic explanation.

At broad spatial scales, climate represents the primary driver governing the distribution of dominant species. Based on high-resolution distribution maps generated via satellite imagery interpretation and field validation across 672 000 grid cells in the Mojave Desert [15], existing research demonstrates that two closely related Joshua tree species, Yucca brevifolia and Y. jaegeriana, occupy largely overlapping aridity niches, but exhibit pronounced geographic divergence driven by discrepancies in seasonal precipitation regimes and annual temperature ranges [15]. At local scales, subsurface geology and soil heterogeneity exert a much stronger control on vegetation persistence than traditionally recognized. Using ground penetrating radar (GPR) and frequency domain electromagnetics (FDEM) in the Negev Desert of Israel, Basson et al. (2023) [16] demonstrated that fracture-hosted micro-aquicludes in weathered chalky bedrock on heterogeneous hillslopes are critical for maintaining shrub drought resilience. By contrast, thick homogeneous soil profiles on homogeneous hillslopes lack water-storage structures and trigger widespread shrub mortality under prolonged drought [16]. Work in the Atacama Desert further supports the role of habitat heterogeneity: moderate aridity zones support the highest soil resource heterogeneity and strongest functional trait differentiation, promoting species coexistence, while both hyper-arid and mesic ends show trait convergence driven by environmental filtering and competitive exclusion, respectively [17].

Traditional diversity research has focused largely on taxonomic diversity, but has increasingly expanded to functional diversity, phylogenetic diversity and endemism. Along the aridity gradient of northern Chile, Poch et al. (2024) found that taxonomic, functional and phylogenetic diversity are highly spatially congruent (local R^2^ > 0.93), all declining with aridity. However, weighted endemism shows only weak spatial correlation with functional and phylogenetic endemism (local R^2^ < 0.48). Desert core regions exhibit higher functional and phylogenetic endemism than expected from taxonomic endemism, reflecting convergent trait evolution and in situ speciation under extreme environmental filtering [18]. Carvajal et al. (2023) further demonstrated scale dependence in assembly mechanisms: trait divergence driven by environmental heterogeneity dominates at the patch scale, whereas stochastic processes prevail at the neighborhood scale [17].

Regarding community stability, Gresse et al. (2026) applied the COMSTAB framework to 13 years of monitoring data from 40 annual plant communities across an aridity gradient in Israel, disentangling three stability drivers for the first time [19]. Asynchrony in species fluctuations emerged as the strongest stabilizing mechanism, operating consistently across all climate zones from arid to Mediterranean, independent of species richness — a finding that challenges the classical insurance hypothesis linking higher richness to greater asynchrony. The averaging effect of species diversity, by contrast, is strongly climate-dependent, peaking in semi-arid and Mediterranean climates and weakening at both arid and mesic extremes [19]. Microhabitat context also modulates stability: open habitats support greater species asynchrony and higher community stability, while shrub understories buffer microclimate and synchronize species fluctuations, reducing overall stability. This contrast is most pronounced in mesic climates; in arid zones, the facilitative effects of shrubs offset the negative consequences of synchrony, consistent with the stress-gradient hypothesis.

In the context of biological invasions, Smith et al. (2023) applied the Detection of Early Season Invasives (DESI) method to Landsat imagery to map invasive annual grasses across the Mojave Desert over 12 years. Getis-Ord Gi analysis identified invasion hotspots covering ~5.4% of the desert, concentrated mainly along the eastern and western climatic transition zones. Random forest modelling ranked soil water-holding capacity (permanent wilting point), soil bulk density, aspect, winter precipitation and elevation as the top predictors of hotspot occurrence, all outweighing anthropogenic variables and highlighting the primacy of soil water availability in desert plant invasions [20–23].

Overall, desert ecology has advanced from aboveground to belowground, from plot-based to regional-scale, and from single-indicator to multidimensional analysis. Nevertheless, key gaps remain: the coupling mechanisms between belowground processes and aboveground communities, cross-system comparisons across bioclimatic desert types, and long-term projections under climate change. Future work should integrate multi-technique, multi-scale and multi-dimensional approaches to advance mechanistic understanding of desert ecosystem responses to global change, and support evidence-based conservation and sustainable management of arid lands.

Presently, several crucial challenges impede progress in this field: (1) Spatial patterns of community structural attributes along elevation, latitude, and longitude have not been systematically quantified in the mountain temperate deserts of Xinjiang, and whether elevational unimodality reflects geometric mid-domain constraints or environmental filtering remains unresolved because species-range data for a formal MDE null model are unavailable. (2) Life-form-specific structural responses of shrubs, semi-shrubs, perennial herbs, and annual herbs to the combined elevational and longitudinal gradients remain unclear. (3) Comparative analyses among the major desert subregions (Altay, Tacheng-Yili, Bortala, Changji-Urumqi, Eastern Tianshan) are insufficient for identifying regional community structural thresholds. (4) The relative importance of elevational versus longitudinal geographic correlates of spatial heterogeneity remains poorly understood, and formal variation partitioning accounting for collinearity among spatial predictors has not been performed.

Based on the aforementioned ecological perspectives and regional environmental characteristics, we formulate three testable statistical hypotheses. Because this study measures community structural attributes rather than species ranges or physiological traits, the hypotheses are framed as expected spatial associations rather than mechanistic confirmations.

Hypothesis 1 (Elevational unimodality): Community structural attributes (height, coverage, density, biomass) exhibit unimodal or parabolic patterns along elevational gradients, with peak values at mid-elevations (700–1100 m) across most subregions and life-forms.

Hypothesis 2 (Longitudinal aridity gradient): Community structural performance decreases from west to east in parallel with the declining westerly precipitation gradient, with the hyper-arid Eastern Tianshan showing the lowest observed community structural attribute values at the regional scale.The community structural attribute values in the eastern sub-region are lower than those in the western sub-regions.

Hypothesis 3 (Life-form structural differentiation): The four life-forms show divergent structural patterns along the combined elevational and longitudinal gradients—shrubs and semi-shrubs maintain relatively stable dominance across gradients, whereas perennial and annual herbs show stronger spatial fluctuations in association with water availability.

Global climate change and intensified human activities have dramatically altered the structure and function of terrestrial grassland ecosystems, especially in arid and semi-arid desert grasslands that are highly sensitive to environmental disturbances. Located in the core hinterland of Central Asia, the temperate desert grasslands in northern Xinjiang are characterized by complex mountain-basin topography, diverse climate conditions, and unique species composition, which form ideal natural conditions for examining how elevational and longitudinal gradients correlate with variation in community structural attributes. In this study, we carried out systematic field sampling across 527 plots and quantitative analysis to address three core questions: (1) Do community structural attributes show unimodal elevational patterns consistent with the MDE expectation, and can these be distinguished from environmental filtering? (2) Is the west-to-east longitudinal gradient associated with consistent declines in vegetation performance across subregions? (3) Do different life-forms (shrubs/herbs) show divergent structural patterns along the combined gradients?

This study describes spatial patterns of community structural attributes across five subregions and four life-forms along three geographic gradients and examines their statistical associations wi th elevational and longitudinal predictors. Results will improve understanding of vegetation community assembly in arid mountain deserts and provide observational reference for conservation planning, though management implications require field validation.

## Materials and methods

### Study area

The latitude zone and continental position of Xinjiang have significantly influenced the formation of its climate, while the region’s undulating mountains and two major basins have led to regional differentiation. In terms of latitude, classified by thermal zones, Xinjiang falls within the temperate zone, with southern Xinjiang belonging to the warm temperate zone and northern Xinjiang to the cold temperate zone. Furthermore, from a land-sea perspective, situated in the heart of the Eurasian continent, Xinjiang is largely unaffected by moist air currents and moisture from the ocean, resulting in its exceptionally high aridity.

No permits were required for this study. The field sampling activities were conducted on publicly accessible sites, and the research did not involve protected species, privately owned land, or regulated biological material that would demand formal approval from environmental or administrative authorities.

The study area is located in the northern desert region of Xinjiang, with a total land area of approximately 4,500 × 10^4^ hm^2^, including 3,000 × 10^4^ hm^2^ of natural grassland. The Tianshan Mountains lie in the central part of Xinjiang, dividing the entire region into two major parts: the northern and southern sections. The area north of the Tianshan Mountains is termed the Northern Frontier, with geographic coordinates ranging from north latitude 41.1°–49.3° and east longitude 79.8°–91.6°, encompassing the Altay Desert Sub-region, the Tacheng-Ili Desert Sub-region, the Bortala Desert Sub-region, the Junggar Desert Sub-region, and the eastern Junggar Desert Sub-region. The landscape of the Northern Frontier is characterized by alternating mountainous terrain and basins; the mountains primarily refer to the Tianshan and Altai Mountains, while the basin is the Junggar Basin. The mountainous areas in northern and western parts of the Northern Frontier are relatively low-lying, allowing substantial moisture and humid air to reach the inland regions of Xinjiang. The Northern Frontier Basin has a relatively low elevation, with an average altitude below 600 m and a minimum point of only 189 m, classified as a semi-enclosed inland basin. Specific climatic conditions include: ① The Junggar Basin has an annual average temperature of 6–8°C; the northern slopes of the Tianshan Mountains, at approximately 2,200 m elevation, reach only 0°C. July exhibits relatively higher average temperatures, being the hottest month of the year, while January is the coldest month. ② The Altai and Tianshan Mountains serve as the primary rainfall centers in Xinjiang, with precipitation increasing as altitude rises. Overall, precipitation in the Northern Frontier decreases gradually from north to south and from west to east. Annual precipitation varies significantly depending on location, with mountainous areas receiving approximately 400–800 mm, basin margins 150–200 mm, and basin centers 100 mm. Snowfall in northern Xinjiang accounts for 20–45% of Xinjiang’s total annual snowfall. In high-altitude regions, snowfall occurs for about 10 months annually except during July–August, with the Tacheng and Ili desert areas experiencing the highest snowfall. The largest snow-covered area in China is the Altay desert region, receiving snowfall for 140–150 days annually, with snow depths reaching 50–60 cm and a maximum of 89 cm. ③ The frost-free period in the basin lasts 150–170 days, while high-altitude areas of the Tianshan Mountains (>3,500 m) have no frost-free days. ④ Annual sunshine duration is long, averaging around 3,000 hours in the basin and slightly lower in mountainous areas, resulting in high total solar radiation. ⑤ The annual accumulated temperature ≥10°C is below 3,000°C in northern Junggar Basin and ranges from 3,000–3,500°C in the south.

The dominant species in the desert vegetation types of this study area include: *Seriphidium transiliense* and *S. borotalalense*; arbor such as *Haloxylon persicum*; and shrubs such as *H. ammodendron*, *Calligonum leucocladum*, *Reaumuria soongorica*, *Anabasis salsa*, and *Nanophyton erinaceum*; perennial plants such as *Kochia prostrata*, *Festuca ovina*, *Stipa sareptana*, and *Carex turkestanica*; and annual plants such as *Ceratocarpus arenarius*, *Salsola collina*, *Salsola nitraria*, *Horaninowia ulicina*, and *Zygophyllum fabago*. In spring, a large number of early-spring short-lived or near-short-lived plants appear, including *Poa bulbosa*, *Ixiolirion tataricum*, *Trigonella arcuata*, *Allium mongolicum*, and *Tulipa iliensis*. In this study, the broad functional-type framework of “shrubs and herbs” is operationalized as four life-forms: shrubs (including small trees such as Haloxylon persicum), semi-shrubs, perennial herbs, and annual herbs.

### Desert area classification

Based on the geomorphological and climatic characteristics as well as grassland types of Northern Xinjiang, the study area is divided into subregions according to the following criteria: First, the classification is based on the differences in endemic constructive species and dominant species among the dominant zonal vegetation types across different areas of the northern Xinjiang desert, together with their adaptation to specific local natural geographical conditions.Second, further account is taken of whether the dominant status of each plant formation varies within the dominant plant formation assemblages of different regions, or whether the addition or reduction of certain plant formations does not alter the basic characteristics of the entire assemblage.Third，the environmental factors affecting grassland growth and the corresponding fundamental grassland types, with climate playing a dominant role in grassland community formation. Since the core of the grassland system consists of “grass” and “livestock,” the classification also considers the characteristics of domestic animal species raised in different grassland regions.Accordingly, the types of management and production activities are also taken as one of the important criteria for delineating typical desert areas in northern Xinjiang, and the administrative boundaries of respective regions are also taken into consideration during the delineation process.Based on field investigations of grasslands in the desert areas of Xinjiang, this study divided the research area in northern Xinjiang into one main region (the desert region of northern Xinjiang) and five subregions for research.(Fig 1).

**Fig 1:**
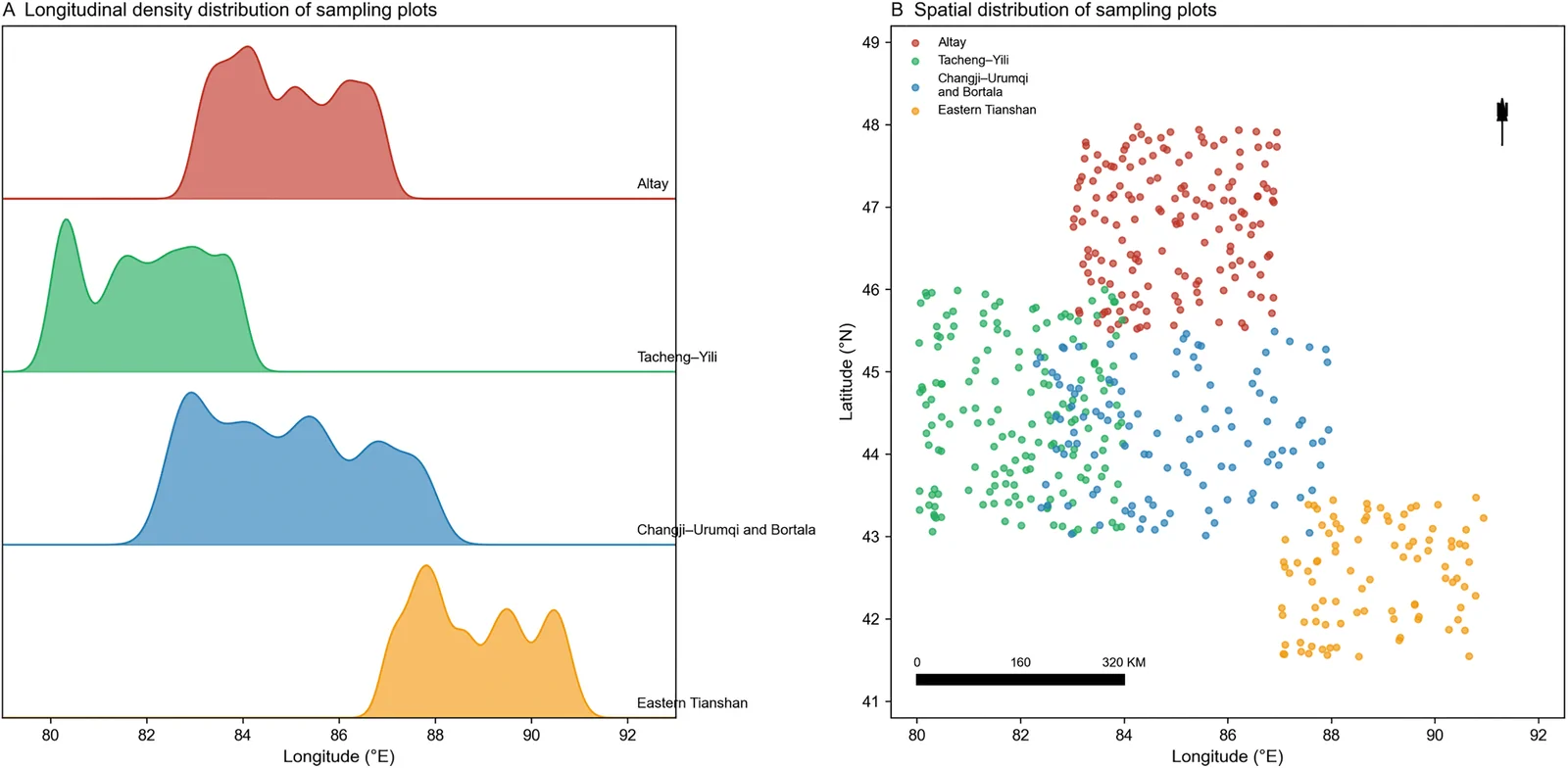
Research area.

(1) The Temperate Arid Zone Desert–Mountain Grassland Region(the desert region of northern Xinjiang)is defined primarily based on zonal climatic characteristics. Main natural characteristics of this region are as follows: The mountain ranges in the northern and western portions of the region are relatively low in elevation, which provides pathways for moist air masses to penetrate inward. The multi-year average annual temperature within the basin ranges from 6–8°C, and the 0°C isotherm is located at an elevation of 2200 meters on the northern slope of the Tianshan Mountains. The multi-year average annual precipitation ranges from 400–600 mm in mountainous areas, with a maximum of up to 800 mm in partial regions, and 150–200 mm at the margins of the basin. This region is approximately located between 40°N and 49°N in latitude.Northern Xinjiang belongs to the temperate arid zone, while southern Xinjiang falls under the warm temperate arid zone. The significant climatic differences stem from the distinct types of arid regions in northern and southern Xinjiang, reflecting variations in latitude and atmospheric circulation influences. The regional boundary runs along the Halke Mountain ridge in the southern Tianshan range, crosses the Nalati–Wukun watershed in the central Tianshan range, extends northeastward to connect with the Bogda Mountain watershed in the eastern Tianshan range, and continues to the low-lying Barikun–Harlake Mountain ridge in the eastern Tianshan range. The area north of this boundary constitutes the “Temperate Arid Zone Desert–Mountain Grassland Region.”The administrative scope of this region encompasses Ili, Tacheng, Altay, Bortala, Changji, Urumqi, as well as counties and cities including Barkol that are under the jurisdiction of Hami Prefecture.
(2) Five subregions: ① The Altay Desert Sub-region (45°00′–49°11′ N, 85°31′–91°04′ E) corresponds to the northern grassland sub-region of the Altai Mountains–Junggar Basin, which is specifically divided into the Haba River-Fuhai County-Fuyun section, the Jeminay Region, and the Qing River-Beita Mountain section, and contains 159 sample plots in total.The predominant livestock species is the meat-altai sheep. Delimited by the north latitude 45° line, this area constitutes a traditional pastoral region where seasonal animal husbandry was first developed. This region is also characterized by relatively abundant forest and water resources in Xinjiang. ② The Tacheng-Ili Desert Sub-region (Tacheng Prefecture: 43°25′-47°15′ North Latitude, 82°16′-87°21′ East Longitude; Ili Prefecture: 42°14′-44°50′ North Latitude, 80°09′-84°56′ East Longitude). The high grazing carrying capacity of grassland resources constitutes a prominent feature of resource utilization in this region.The predominant livestock species are Ili horses and Xinjiang fine-furred sheep, making this region the best pastoral area in Xinjiang. Tacheng-Ili Desert Sub-region comprises 164 sample plots. ③ The Bortala Desert Sub-region (44°02′N – 45°23′N, 79°53′E – 83°51′E) covers the Bortala Mongol Autonomous Prefecture, including Bole, Jinghe, and Wenquan counties, characterized by inte-rmountain basins and strong wind conditions.The annual average precipitation in the plain region of this sub-region ranges from 90 mm to 190 mm. In mountainous areas with an altitude between 1500 m and 2000 m, the annual average precipitation is below 300 mm, and precipitation shows an increasing trend with the rise of altitude. The dominant grassland types in this area are desert grassland and steppe grassland. ④ The Changji-Urumqi Desert Sub-region (43°00′N – 45°38′N, 85°17′E – 90°32′E) encompasses the mid-section of the Tianshan northern slope to the southern grassland area of the Junggar Basin.In this desert sub-region, grassland resources exhibit distinct north-south heterogeneity, accompanied by evident zonal variations from west to east, which are most prominently reflected in humidity: aridity gradually increases from west to east. These inherent natural conditions directly determine the grassland types and vegetation community characteristics in different geographic units of the sub-region.Specifically includes: Wusu-Urumqi zone and Miquan-Mulei zone (Extending from Wusu in the west to Mulei in the east, this vast region encompasses the mountainous areas along the northern slope of the Tianshan Mountains and the southern part of the Junggar Basin, spanning from the foothill plains to the desert, and is located south of 45° north latitude), where agriculture and animal husbandry are integrated.Changji-Urumqi and Bortala Desert Sub-region comprises 110sample plots. ⑤ The East Tianshan Desert Sub-region (It is mainly desert grassland in Hami area, located at approximately 40°43′–45°05′ North Latitude and 90°08′–96°23′ East Longitude) encompasses the mountainous areas and intermountain basin grasslands along the eastern section of the Tianshan northern slope, specifically including the Barkol–Yiwu area (The area between the easternmost point of the northern slope of the Tianshan Mountains and the easternmost point of the southern slope of the Altai Mountains, including the Barkol–Yiwu intermontane basin; this region is primarily dedicated to the rearing of Barkol horses and features a pastoral-based agricultural system integrated with forestry activities.) and the oasis grassland sub-region at the southern edge of the Junggar Basin.East Tianshan Desert Sub-region comprises94sample plots.

### Experiment design and field investigation

The research conducted desert field surveys at 527 sampling plots during the plant growing season in mid-May, July and September each year over a three-year period.Plot distribution across the five subregions was as follows: Altay (n = 159), Tacheng-Yili (n = 164), Bortala and Changji-Urumqi (n = 110), and Eastern Tianshan (n = 94); sample sizes were uneven because plots were selected purposively to represent dominant habitat types within each subregion, and accessibility varied substantially across the remote desert landscape. The minimum distance between adjacent plots was approximately 50 m to reduce spatial autocorrelation, though formal spatial independence was not tested at the design stage. Select four typical 100-m-long sample strips in each area, with a minimum spacing of 50 m between them; place one 1 m × 1 m sample plot at every 25-m interval. Observe and record the quantitative characteristics of the plant above-ground biomass; for the plant below-ground biomass, perform stratified root sampling along the diagonal axis of the plot using a root drill (diameter 9 cm), soil drill (diameter 5 cm), or ring knife (diameter 5 cm) at fixed depths of 0–5 cm, 5–10 cm, 10–20 cm, 20–30 cm, 30–50 cm, 50–70 cm, and 70–100 cm, respectively.

All field surveys were conducted within a single growing season (May–September) to eliminate the confounding effects of inter-annual climatic variation. No permanent plots were resurveyed across years, and thus temporal replication was not incorporated in this study. A nested quadrat sampling design was employed to investigate the quantitative characteristics of plant communities in the Northern Frontier Desert (Fig 2). Each sampling plot consisted of one 10 m × 10 m quadrat for woody shrub species, with five systematically arranged 1 m × 1 m subquadrats established for herbaceous vegetation surveys. During field sampling, topographically flat and vegetationally representative sites were prioritized to reflect regional plant community features. A total of 30 replicated small quadrats were measured for areas with homogeneous terrain, while additional quadrats were deployed for topographically complex areas to ensure sampling completeness and representativeness.

**Fig 2:**
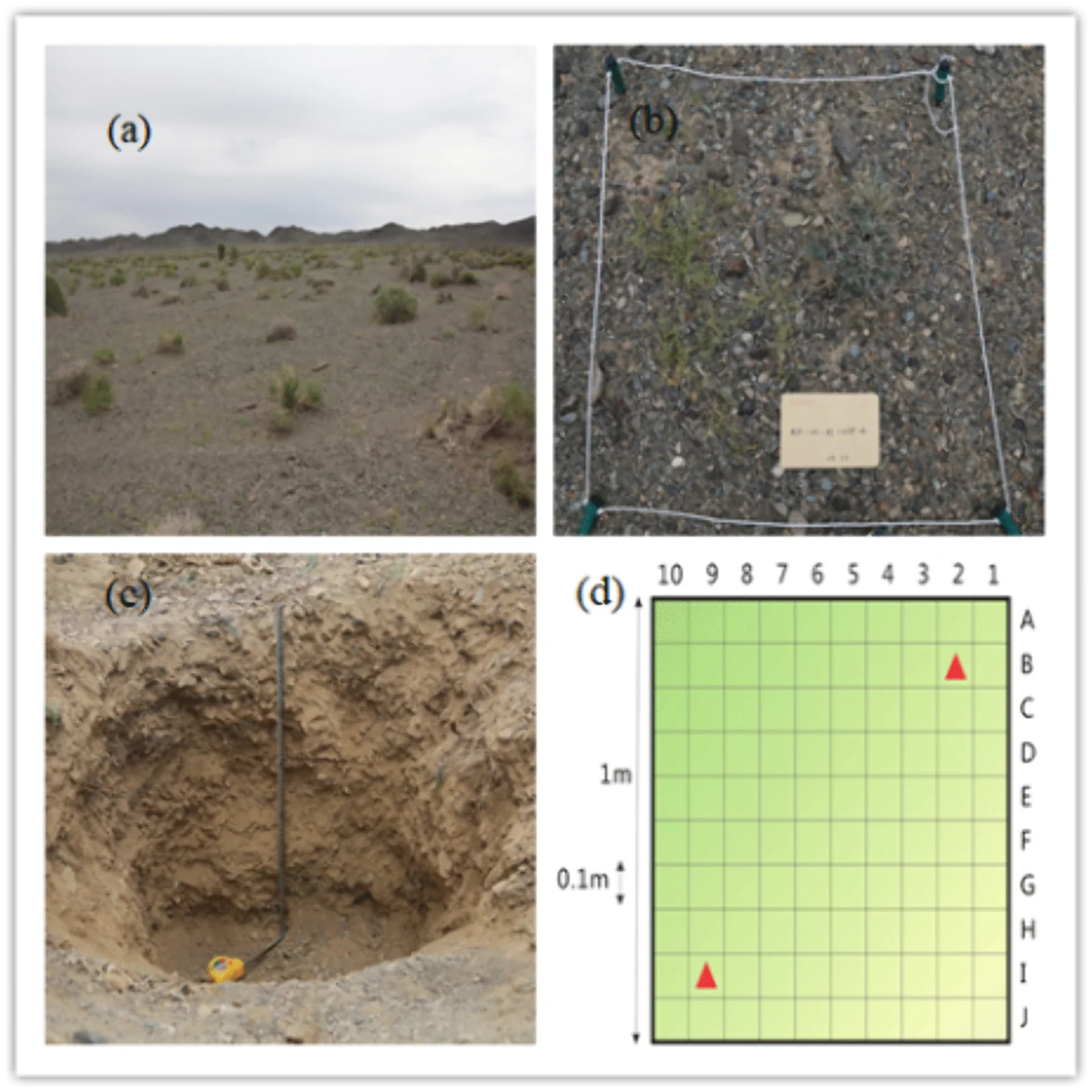
Quadrat design and soil profile.

The composition of plant life forms in the Xinjiang desert is distinctive and highly complex. Communities with more intricate synusial structures are all distributed in the Junggar Basin.Among the gymnosperms of the Xinjiang desert, super-xerophytic evergreen leafless shrubs represent a key constructive life form of the local shrub desert, and are capable of forming synusia in other desert types as well.Although super-xerophytic evergreen broad-leaved shrubs play a limited role in the Xinjiang desert, they can develop synusia and constitute one piece of evidence for the antiquity of this desert region.Super-xerophytic summer-green broad-leaved shrubs are the formative components of the Xinjiang shrub desert, and some species can form distinct dominant synusia.Semi-shrubs also make up a vital part of the Xinjiang desert and serve as a crucial life form defining the desert’s characteristics. For instance, summer-green microphyllous semi-shrubs, summer-green clavate-leaved semi-shrubs and summer-green succulent-leaved semi-shrubs can all form synusia, with many species developing into constructive synusia.Perennial herbs are able to form synusia in the Xinjiang desert, but cannot give rise to constructive synusia. Perennial ephemeral plants and annual herbs both play distinctive roles in desert communities.

The survey encompassed four plant life-forms: shrubs (including small trees), semi-shrubs, perennial herbs, and annual herbs. For each quadrat, species composition was fully documented, and multiple community structural indices were measured with standardized protocols. Plant natural growth height was quantified using a tape measure at a precision of 0.1 cm. Community coverage was estimated via a combined visual and grid method with a precision of 1%, and all estimations were performed consistently by two fixed observers to minimize inter-observer bias; needle-poking method was additionally adopted to verify species coverage. Plant density was uniformly defined as the number of living individuals per square meter (individuals/m^2^) across all life-forms.

For aboveground biomass quantification, For each quadrat, the aboveground living parts of plants of each species were clipped at ground level. The fresh biomass of living plants was measured (g/m^2^). After clipping all herbaceous species within the quadrat, surface litter and standing dead materials were collected as litter samples, weighed (g/m^2^), placed into labeled bags, and transported back to the laboratory. The samples were wrapped with paper, subjected to oven-killing at 105 °C for 30 min, and then dried continuously at 80 °C for 24 h, followed by measurement of dry biomass (g/m^2^). Density as number of living individual plants per unit area (individuals m^-2^)” for all life-forms.For each surveyed quadrat, community coverage (%) was estimated via the visual estimation method, and plant cluster height (cm) and density (individuals/m^2^) were quantified via direct measurement.Furthermore, Surface litter and standing dead plant materials were collected and weighed separately in this study; these components were excluded from the above-ground living biomass data-set. In addition, GPS was used to record the longitude, latitude and elevation of each sampling site. Meteorological data from meteorological stations of Xinjiang Meteorological Bureau, Xinjiang Statistical Yearbook and other datasets, together with the longitude, latitude and elevation of field sampling sites, were adopted to calibrate the data of sampling points via interpolation. Up to now, the field sampling campaign for typical desert vegetation and soils in northern Xinjiang is still ongoing. A typical sampling method was applied for plot surveys to sample plant types and quantitative community characteristics within the plots.

The classification of grassland plant ecological-economic groups follows the system proposed by Xu Peng (1994), which is based on similarities in ecological-biological characteristics and forage value and is widely used in grassland management in China. The Artemisia desert plants on the northern slope of the Tianshan Mountains are classified into four groups: shrubs, semi-shrubs, perennial herbs, and annual herbaceous plants. Shrubs are defined as woody plants with persistent aboveground stems and height > 50 cm at maturity; this group includes small trees such as Haloxylon persicum, which, despite being technically a small tree, is treated as a shrub in this classification because its growth form, height range and ecological role in the desert community are functionally equivalent to shrubs. Semi-shrubs are woody-based plants with herbaceous upper shoots that die back annually (e.g., Seriphidium species). Perennial herbs are non-woody plants that persist for multiple years, and annual herbs complete their life cycle within a single growing season. This classification system was chosen because it aligns with standard grassland survey protocols in Xinjiang and enables comparison with previous regional studies.

Guided by theories associated with grassland resource surveys, community diversity, and plant taxonomy, this study applies traditional field vegetation sampling techniques in combination with modern instrumentation, with a focus on a typical desert area in northern Xinjiang. It integrates field sampling, monitoring, and analytical identification to explore the spatial distribution patterns of community structural attributes and biomass. The field survey was carried out in two stages: The first stage involved surveys of desert vegetation in the eastern parts of the Junggar Basin, including Mulei in the eastern Tianshan, and Barkol County, Yizhou District, Gaochang District, and Toksun County in the eastern desert sub-region of the eastern Junggar; The second stage covered surveys across the Junggar Basin, its northern and western regions, including Urumqi City, Hutubi County, Fukang City, Qitai County, Karamay City, Tuoli County, Yumin County, Tacheng City, Jinghe County, Bole City, Wenquan County, Yining County, Gongliu County, Xinyuan County, Emin County, Jimunai County, Habah River County, Burqin County, Altay City, Fuhai County, Fuyun County, and Qinghe County.

### Data statistics and analysis

Studies on pairwise interspecific association mainly focus on two aspects: (1) applying $\chi^{\text{2}}$ statistical tests to determine whether an association exists between two species; (2) calculating association indices to quantify the magnitude of pairwise interspecific association.The overall inter-specific association among species in the plant community was assessed using the variance ratio (VR) approach proposed by Schluter (1984). A presence–absence matrix was constructed from the occurrence of each species across quadrats, and the VR was computed to quantify the overall strength of inter-specific association. Under the null hypothesis that all species are independently distributed with no association among one another, a VR value of 1 indicates acceptance of the null hypothesis, i.e., no significant inter-specific association. A VR > 1 leads to rejection of the null hypothesis, indicating an overall positive inter-specific association among species, whereas a VR < 1 indicates an overall negative inter-specific association. The statistical significance of the observed $\chi^{\text{2}}$ was then tested accordingly.

Overall association test.A null hypothesis was first established, assuming that all species are independent of one another and that no significant overall inter-specific association exists. The VR value was calculated using the following formula:


$$\begin{array}{c} VR={S_{T}}^{\text{2}}/{\delta^{\text{2}}}_{T} \end{array}$$

$$\begin{array}{c} \delta^{\text{2}}T=\sum_{i=1}^{s}P_{i}\text{\textasciitilde{}}(\text{1}-P_{i}),\text{\textasciitilde{}}S_{T}^{2}\;=(\text{1}/N)\text{\textasciitilde{}}\sum_{i=1}^{N}(T_{j}-t{)}^{\text{2}} \end{array}$$

$$\begin{array}{c} \chi^{\text{2}}=\sum \frac{{\left(\text{observed value-estimated value}\right)}^{\text{2}}}{\text{estimated value}} \end{array}$$

where S is the total number of species, N is the total number of quadrants, n_i_ is the number of quadrants where species i occurs, P_i_ is the frequency of species i, T_j_ is the total number of species recorded in quadrant j, t is the mean number of species across all quadrats, S_t_ is the variance of species number across all quadrats, and $\delta_{T}$ is the variance in occurrence frequency for all species.

Elevation, latitude, and longitude represent fundamental spatial coordinates that typically exhibit strong correlations and significant spatial auto-correlation. To eliminate the analytical biases caused by multicollinearity among multiple variables, this study independently analyzed the ecological effects of each individual spatial gradient.First, the response patterns of quantitative community characteristics and regional differences were investigated. Regression models were established separately for five study regions. Quadratic polynomial regression was fitted to four functional groups (shrubs, sub-shrubs, perennial herbs, and annual herbs) and four community traits (height, coverage, density, and biomass) along three spatial gradients (elevation, latitude, and longitude). The sign of the regression coefficients was used to identify ecological response patterns, including linear increasing trends, linear decreasing trends, unimodal patterns (rising first and then falling), and U-shaped patterns (falling first and then rising). The gradient threshold values corresponding to the inflection points of each response pattern were further calculated.Second, gradient sensitivity ranking was performed for desert vegetation life forms. All variables were standardized prior to analysis, and standardized beta regression coefficients were calculated. Combined with the coefficient of variation (CV = standard deviation/ mean) of functional traits along each gradient, this approach quantitatively assessed the sensitivity of different desert vegetation life forms to identical spatial gradients, as well as the differential responses of the same life form to distinct spatial gradients.At this stage, other members of the research group are still engaged in field data acquisition of desert vegetation and soil in northern Xinjiang.

## Results

### Composition of main species and analysis of overall inter-specific association in the northern Xinjiang desert

Deserts account for 46.9% of the total grassland area in Xinjiang, representing an important grassland resource for the region; approximately 40% of these desert grasslands are utilized for raising domestic livestock (primarily cattle and sheep). The development of species, the stratification structure, and the species composition within the northern Xinjiang desert zone are adapted to the seasonal climatic dynamics. This study selected two community layers from the temperate desert of northern Xinjiang—the herbaceous layer and the shrub layer—comprising a total of 66 herbaceous species and 22 shrub species. The dominant species include *Seriphidium borotalensis, S. transiliense, S. terrae-albae, Anabasis salsa, Nanophyton erinaceum, Haloxylon persicum, H. ammodendron, Reaumuria soongorica*, among others (Tables 1 and 2).

**Table 1:** Name and abbreviation of main species in herbaceous layer in Northern Xinjiang.

ABBREVIATION	SPECIES SCIENTIFIC NAME	ABBREVIATION	SPECIES SCIENTIFIC NAME
*A*	*Anabasis salsa*	*Sv*	*Setaria viridis*
*Ne*	*Nanophyton erinaceum*	*Ap*	*Atriplex patens*
*Cl*	*Ceratoides latens*	*An*	*Anabasis elatior*
*Ar*	*Artemisia*	*Aq*	*Astragalus*
*Kp*	*Kochia prostrata*	*Ay*	*Anabasis aphylla*
*Ci*	*Ceratocarpus utriculosus*	*Ag*	*Artemisia gracilescens*
*Hg*	*Halogeton glomeratus*	*L*	*Leymus racemosus*
*Ph*	*Peganum harmala*	*Is*	*Anabasis salsa*
*Rs*	*Reaumuria kaschgarica*	*Ac*	*Atriplex cana*
*St*	*Stipa capillata*	*Sk*	*Seriphidium terrae-albae*
*Sl*	*Salsolaabrotanoides*	*Ep*	*Eragrostis pilosa*
*Ps*	*Petrosimonia sibirica*	*Aa*	*Artemisia arenaria*
*Tt*	*Tribulus terrester*	*Hu*	*Horaninowia ulicina*
*Ce*	*Chenopodium album*	*Ls*	*Limonium sinense*
*Ha*	*Haloxylon ammodendron*	*Am*	*Allium mongolicum*
*Ch*	*Corispermum hyssopifolium*	*Sc*	*Salsola collina*
*Ai*	*A.laterifllorum*	*Fo*	*Festuca ovina*
*Sr*	*Salsola ruthenica*	*S*	*Seriphidium transillense*
*Pt*	*Aristida pennata*	*So*	*Sophora alopecuroides*
*Ra*	*Ephedra distachys*	*Ae*	*Aeluropus littoralis*
*Ed*	*Ephedra intermedia*	*Hs*	*Halocnermum strobilaceum*
*Kf*	*Kalidium foliatum*	*Hh*	*Heteropappus hispidus*
*Pa*	*Phragmites australis*	*Ka*	*Karelinia caspia*
*Ca*	*Carex* sp.	*Cr*	*Chenopodium aristatum*
*Lr*	*Iljinia regelii*	*Ti*	*Stipa caucasica Schmalh*
*Ab*	*Anabasis brevifolia*	*Au*	*Plantago lessingii*
*Ct*	*Convolvulus tragacanthoides*	*As*	*Alhagi sparsifolia*
*Cs*	*Cleistogenes squarrosa*	*Sb*	*Seriphidium borotalense*
*Gl*	*Stipa glareosa*	*Sp*	*Salsola brachiata*
*Nk*	*Seriphidiumsantolinum*	*Sg*	*Suaeda dendroides*
*Ug*	*Seriphidium gracilensces*		
*Eo*	*Eremopyrum triticeum*		
*Co*	*Ceratocephalusorthoceras*		
*Pl*	*Poa annua*		
*Sa*	*Salsola arbuscula*		
*Dn*	*Draba nemorosa*		

**Table 2:** Name and abbreviation of main species in shrub layer in Northern Xinjiang.

ABBREVIATION	SPECIES SCIENTIFIC NAME	ABBREVIATION	SPECIES SCIENTIFIC NAME
*Cl*	*Ceratoides latens*	*Sa*	*Salsola abrotanoides*
*Ag*	*Artemisia gracilescens*	*Ns*	*Nitraria sphaerocarpa*
*Ha*	*Haloxylon ammodendron*	*As*	*Alhagi sparsifolia*
*Af*	*Atraphaxis frutescens*	*Hp*	*Haloxylon Persicum*
*Cm*	*Calligonum mongolicum*	*Sr*	*Salsola arbuscula*
*Kf*	*Kalidium foliatum*	*Ca*	*Caragana*
*Ep*	*Ephedra przewalskii*	*Hs*	*Halocnermum strobilaceum*
*Ir*	*Iljinia regelii*	*Cr*	*Calligonum rubicundum*
*An*	*Anabasis brevifolia*	*Sp*	*Sueada physophora*
*Es*	*Ephedra sinica*	*Rs*	*Reaumuria soongorica*
*Zx*	*Zygophyllum xanthoxylum*		
*Hc*	*Halostachys caspic*		

The overall interspecific association was analyzed for representative dominant species comprising 66 herbaceous and 22 shrub taxa selected based on high importance values (e.g., abundance and frequency). Most plant species exhibit a clumped spatial distribution, while regular distribution is rarely detected. The type of spatial distribution pattern is generally determined by local ecological conditions and inter-specific interactions with coexisting species [24].

An overall inter-specific association analysis was conducted based on the 66 representative herbaceous species and 22 shrub species, selected for high abundance and frequency. The spatial distribution patterns of most plant species exhibit a clustered distribution, with regular distribution being rare; this typically reflects the patchy distribution of suitable habitats and is also linked to interactions among co-occurring species. To assess the degree of overall co-occurrence, we used the variance ratio (VR) method, which tests the null hypothesis that species associations are independent. If VR > 1, the null hypothesis of independence can be rejected in favor of overall positive association; if VR < 1, an overall negative association is indicated. The significance of VR was evaluated using the test statistic W = VR × N, where n is the number of plots, compared against a chi-square distribution. The calculations for the dominant species communities in the herbaceous and shrub layers are presented in Table 3. For both the herbaceous layer and the shrub layer, VR > 1 indicates an overall positive association among species pairs; however, the test statistic W fell within the non-significant range (*χ*^2^_0.95*N*_ < *W* < *χ*^2^_0.05*N*_ P > 0.05), meaning that the positive association was not statistically distinguishable from random variation..A non-significant positive association does not demonstrate community stability or maturation; it merely indicates that significant negative (competitive) associations were not detected, which could result from diffuse interactions, or the snapshot nature of the sampling. This analysis is descriptive and is not directly connected to the three principal Hypothesiss regarding elevational and longitudinal gradients.

**Table 3:** Overrall interspecific associations among main plant populations of different hierarchical levels of temperete desert grassland community in northern zone of Xinjiang.

Levels	VR	Test statistics W	χ^2^ (χ^2^_0.95_，χ^2^_0.05_)	Results
herb layer	1.02	3893.56	(3688.17，3976.1)	Non-significant positive correlation
shrub layer	1.03	2592.59	(2388.75，2621.52)	Non-significant positive correlation
Herbaceous layer and shrub layer	1.01	4405.52	(4221.3，4528.97)	Non-significant positive correlation

### Spatial patterns in the Altay desert region

#### Elevation gradients.

As shown in Fig 3, shrub and semi-shrub height showed unimodal patterns, peaking at 800–1000 m. Perennial herb height increased with elevation, whereas annual herb height decreased. Shrub coverage peaked at 800 m and decreased significantly above 1000 m. Semi-shrub coverage exhibited an upward-opening parabolic pattern. Perennial herb coverage increased with elevation, and annual herb coverage peaked at 800 m. Shrub and semi-shrub densities were unimodal with maxima at 800 m. Annual herb density was highest at 600 m and decreased sharply above 1000 m. At an altitude of 800 m, the biomass of shrubs attains its peak value. As the altitude ascends, the biomass of shrubs demonstrates a downward trend. The biomass of annual herbaceous plants follows a similar pattern to that of shrubs. Conversely, the biomass of perennial herbaceous plants rises with the increasing altitude. These unimodal elevational patterns in shrub height, coverage, and density are descriptively consistent with a mid-elevation optimum, which could reflect either geometric mid-domain constraints or environmental filtering (optimal hydrothermal conditions); the present data cannot distinguish these mechanisms.

**Fig 3:**
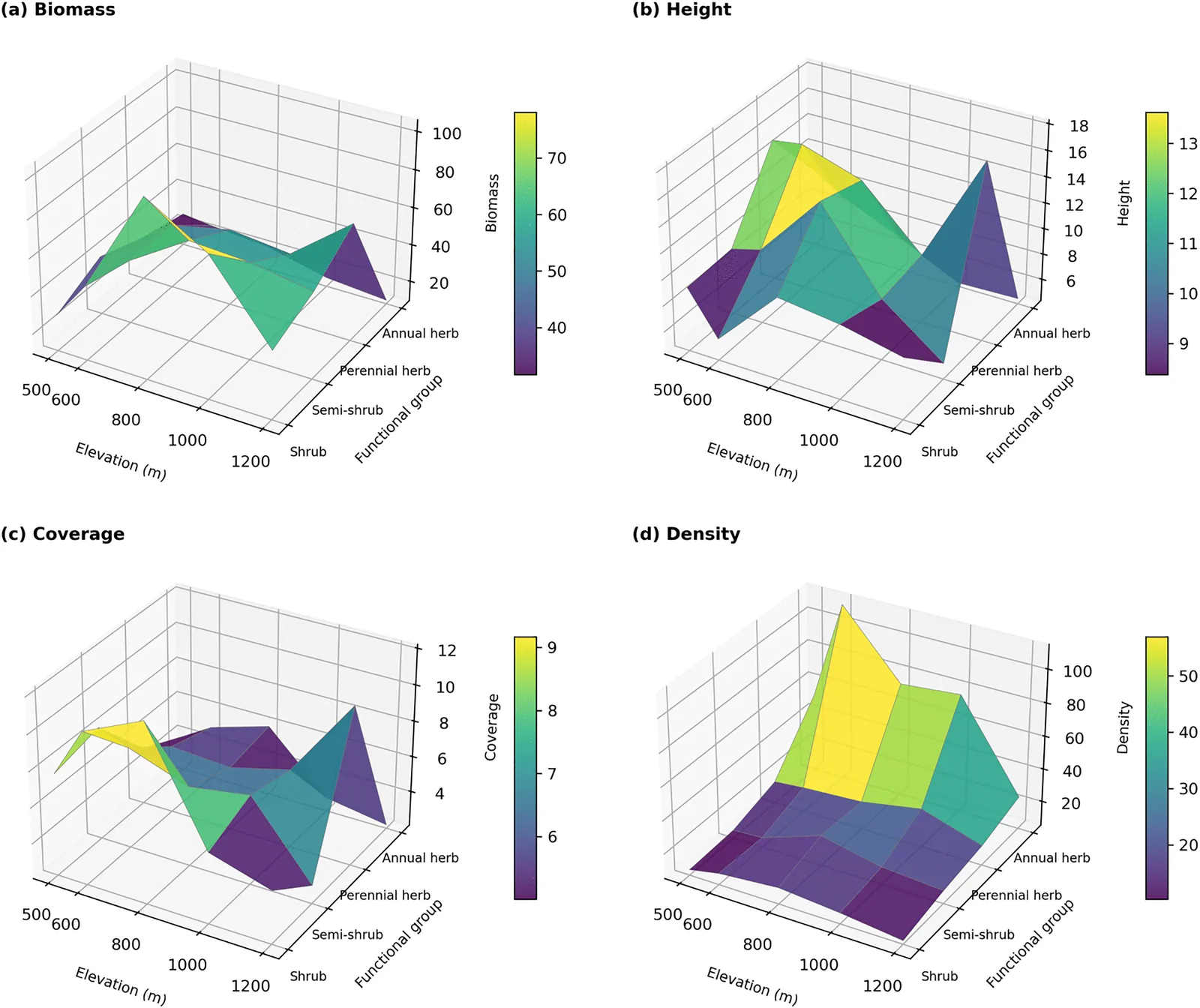
Spatial distribution of plant ecological-economic groups and altitude in the Altay Desert.

#### Latitude gradients.

Fig 4 shows that in the desert grassland of the Altay region, the height of shrubs, semi-shrubs, and annual herbs gradually increases with increasing latitude. In contrast, the height of perennial herbs exhibits a pattern of first decreasing and then increasing with latitude. Shrub cover shows an initial increase followed by a decrease with latitude, reaching a maximum value of 16 at approximately46°- 47°N. Semi-shrub cover generally exhibits a significant decreasing trend with latitude, approaching its maximum near 45°N and then decreasing markedly with each 1° increase in latitude. Perennial herb cover shows a non-significant trend of first decreasing and then increasing with latitude. Annual herb cover gradually increases with latitude, also with a non-significant trend; (vegetation) cover reaches its minimum at approximately 45°N and attains its maximum at approximately 48°N.Shrub density generally exhibits a unimodal (inverted U-shaped) pattern with latitude, peaking at approximately 47°N. In contrast, semi-shrub density shows an opposite trend to that of perennial and annual herb density with latitude: semi-shrub density first decreases and then increases, but with an overall decreasing trend. Annual herb density first increases and then decreases, whereas perennial herb density shows an overall increasing trend with latitude. Shrub biomass reaches its maximum at 47°N and then decreases with increasing latitude. Semi-shrub biomass exhibits an overall decreasing trend with increasing latitude.

**Fig 4:**
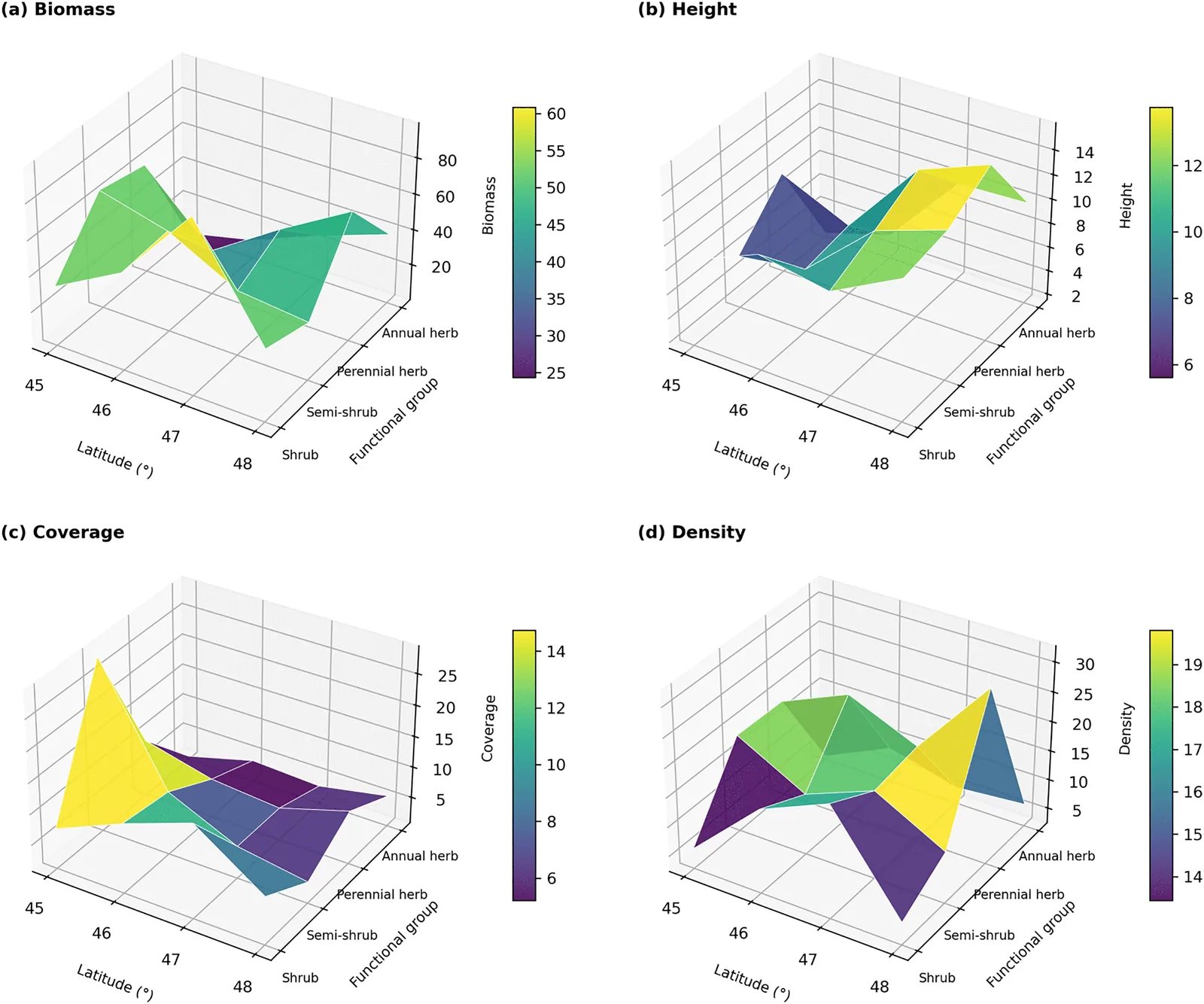
Spatial distribution of ecological-economic groups of desert plants in Altay by latitude.

#### Longitude gradients.

As shown in Fig 5, with increasing longitude, the average height of shrubs presents a trend of decreasing first and then increasing, and the minimum value of the average height is recorded between 86°E and 87°E. The average height of semi-shrubs exhibits a consistent downward trend as longitude increases, with the maximum mean height observed between 84°E and 85°E.Perennial herb height exhibits a trend similar to that of shrub height. Annual herb height shows a slight increase near 86E, followed by a marked decrease at 90E. Between 87 and 88E, shrub height, semi-shrub height, and perennial herb height all increase notably compared to their values between 86 and 87E. With increasing longitude, shrub and semi-shrub cover display a first-decrease-then-increase pattern, with semi-shrub cover increasing significantly. Perennial herb cover follows a trend similar to that of shrub cover. Annual herb cover initially increases and then decreases; at approximately 90E, it declines compared to the highest cover value, which occurs near 87E. Shrub density and semi-shrub density exhibit parabolic (unimodal) changes with longitude. Semi-shrub density increases markedly between 84 and 87E. Perennial herb density and annual herb density exhibit similar trends, both showing an initial increase followed by a decrease. Between 84 and 89E, annual herb density is the highest among shrub, semi-shrub, and perennial herb densities, and a maximum value at 87E. At 90E, annual herb density decreases significantly. Shrub biomass reaches its highest value at 86E and then decreases markedly with increasing longitude. Semi-shrub biomass shows relatively little overall change and remains at relatively low values. Perennial herb biomass and annual herb biomass exhibit similar trends with longitude. The eastward decline in annual herb cover and density toward 90E is descriptively consistent with a longitudinal gradient, which may correspond to increasing aridity, though direct soil moisture measurements were not obtained to confirm this mechanism.

**Fig 5:**
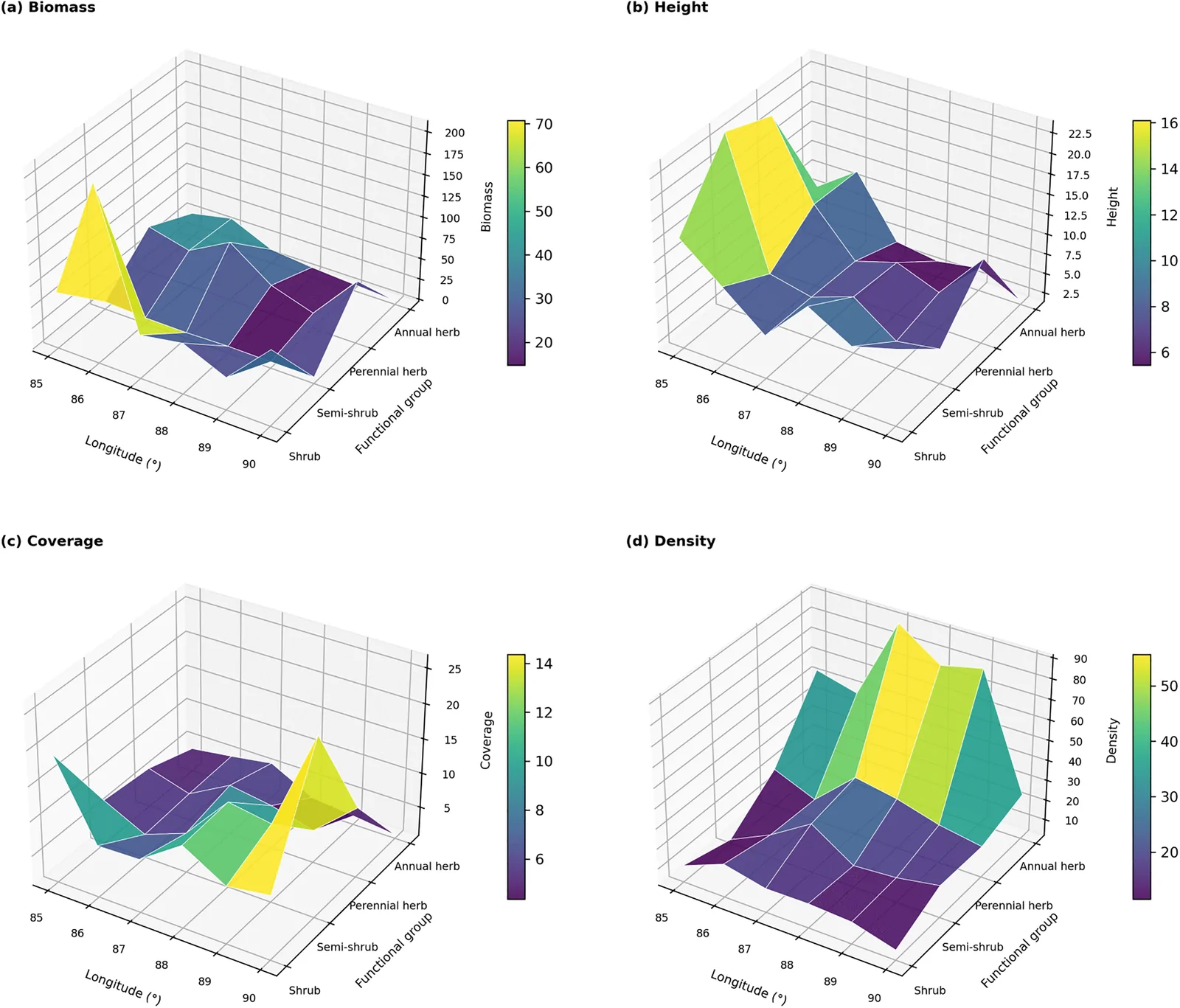
Spatial distribution of ecological-economic groups and longitude values for desert plants in Altay.

### Spatial patterns in the Tacheng-Yili desert region

#### Community characteristics and longitudinal spatial distribution in the Tacheng Desert.

Fig 6 shows that with changing longitude, shrub height exhibits a unimodal (single-peak) pattern, with the lowest value at 85°E, which is significantly lower than the maximum recorded at 83°E. Semi-shrub height first decreases and then increases. Perennial herb height gradually increases with increasing altitude. The height of annual herbs does not present a significant changing trend between 82°E and 85°E, while it decreases significantly around 86°E. Regarding cover, shrub cover and perennial herb cover follow essentially the same pattern—initially increasing and then decreasing—both peaking at 84°E. Semi-shrub cover gradually increases with longitude, reaching its maximum at 86°E. Annual herb cover exhibits an overall decreasing trend; at 86°E, its cover is markedly lower than the maximum value observed at 82°E. For density, shrubs, semi-shrubs, and perennial herbs all show a similar unimodal trend of first increasing and then decreasing with longitude. In contrast, annual herb density decreases significantly; at 86°E, it is substantially lower than the maximum density recorded at 82°E. Shrub biomass is higher at 82°E and 84°E than at other longitudes. Biomass values of semi-shrubs, perennial herbs, and annual herbs are all consistently low.

**Fig 6:**
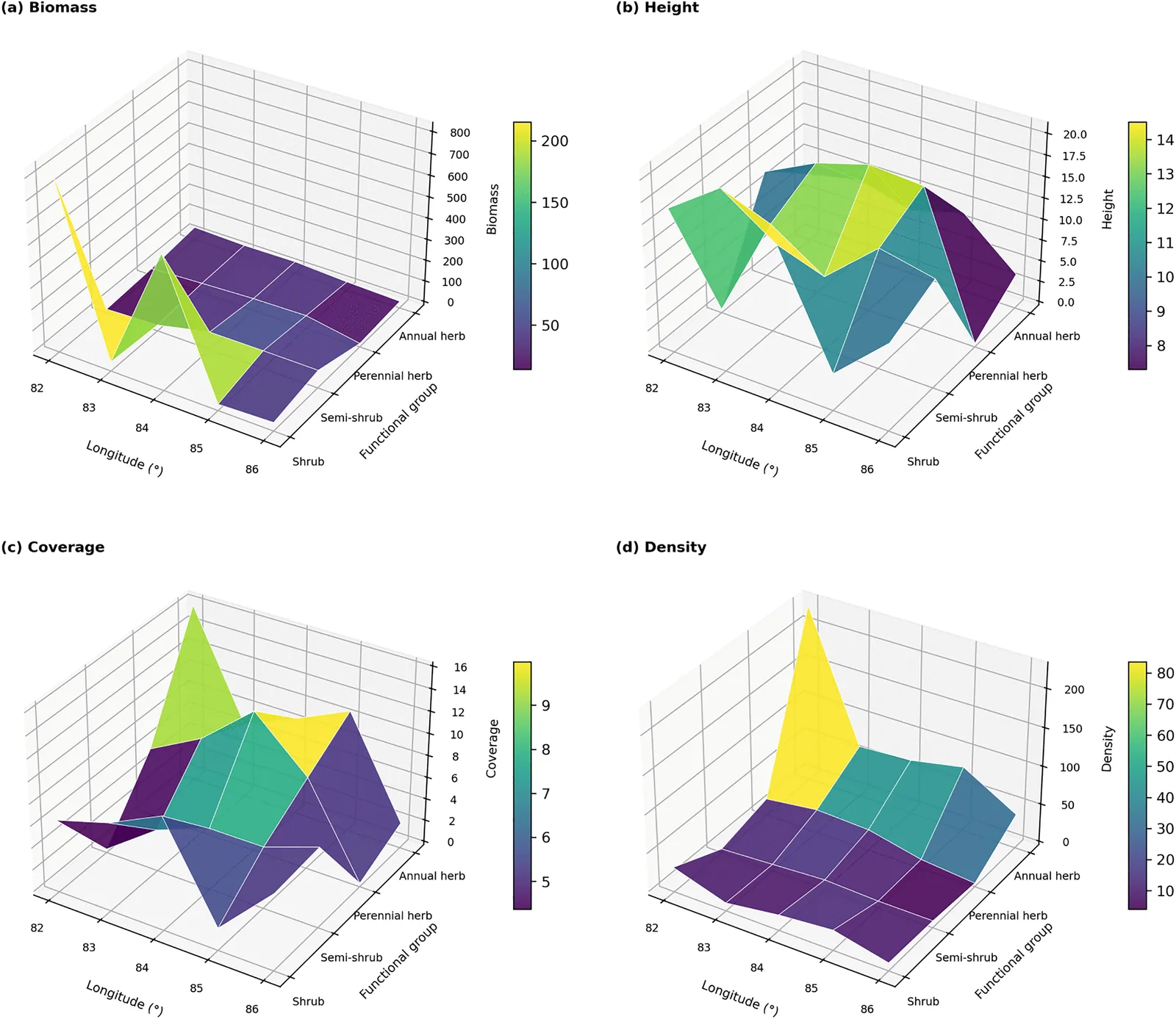
Plant ecological-economic groups and their longitudinal spatial distribution in the Tacheng Desert Region.

#### Community characteristics and latitudinal spatial distribution in the Tacheng Desert.

Fig 7 shows that with increasing latitude, the heights of shrubs, perennial herbs, and annual herbs follow a unimodal (first increase then decrease) pattern, all peaking at 45°N. In contrast, semi-shrub height exhibits a trend of first decreasing and then increasing. At 46°N, both shrub height and perennial herb height are significantly lower than at 45°N and 44°N. Regarding cover, shrubs, semi-shrubs, perennials, and annuals all show broadly similar trends, following a unimodal curve with latitude.Specifically, the cover of annual herbs at 44°N is higher than that at 43°N; in contrast, the cover of shrubs and perennial herbs at 44°N decreases as latitude increases by 1°.In terms of plant density, shrub density, perennial herb density and annual herb density all show a U-shaped (concave-up parabolic) distribution pattern along the latitudinal gradient, among which the variation in annual herb density is the most significant.Shrub biomass reaches its maximum at 44°N and then declines markedly with increasing latitude. By contrast, perennial herb biomass and annual herb biomass show no significant changes across the latitudinal gradient.

**Fig 7:**
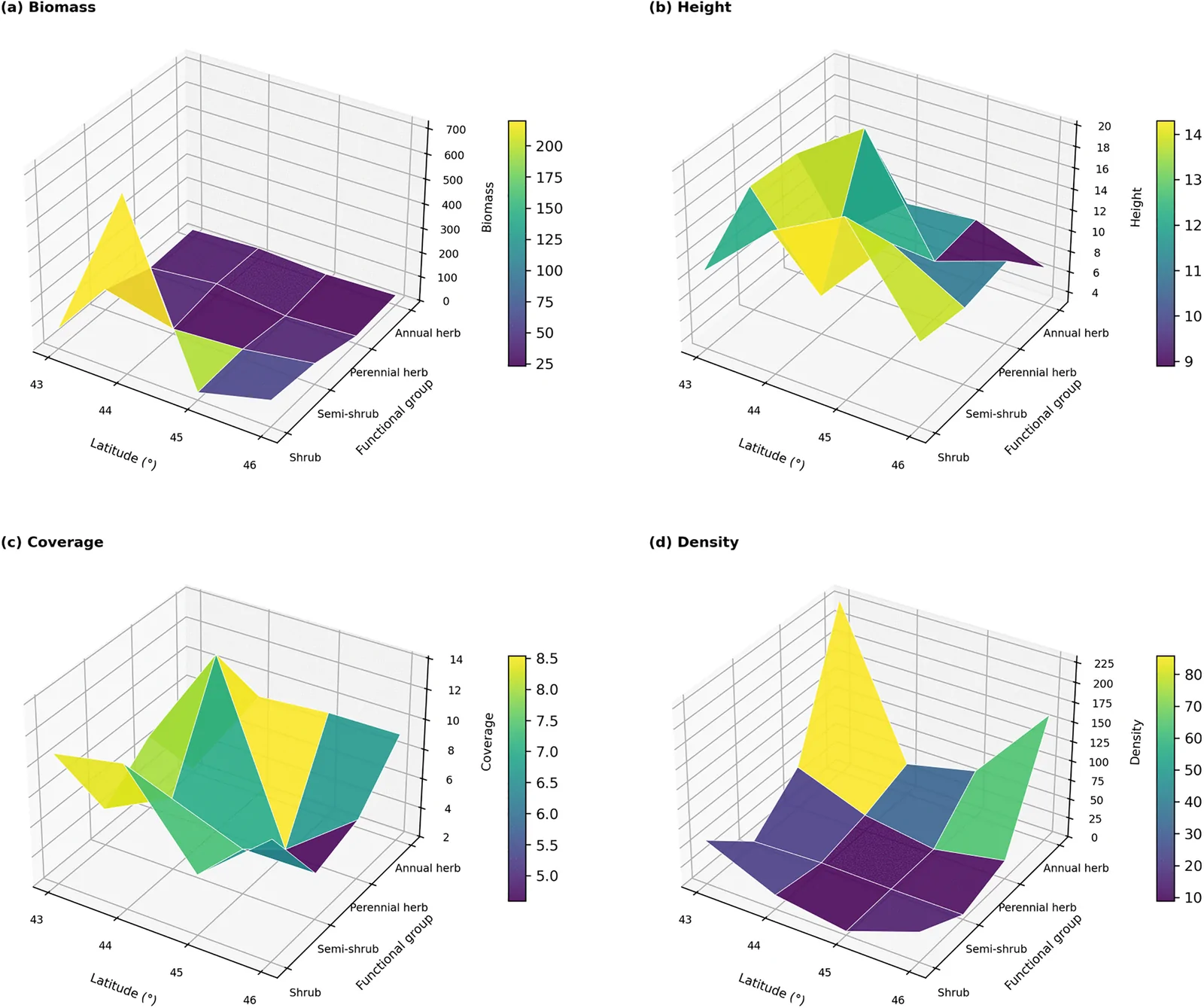
Spatial variation of plant eco-economic groups and latitude in Tacheng Region.

#### Community characteristics and altitudinal spatial distribution in the Tacheng Desert.

Fig 8 shows that with increasing altitude, semi-shrub height follows a unimodal (parabolic) pattern, first increasing and then decreasing. The minimum height occurs at 1100 m, where it is significantly lower than at 500 m. Between 300 m and 800 m, annual herb height shows no significant change, but gradually decreases above 800 m. Perennial herb height exhibits a pronounced decreasing trend above 800 m. With the increase in altitude, the cover of perennial herbs shows a gradual increasing trend, while it decreases sharply at altitudes above 1100 m. Within the altitude range of 300 m to 1100 m, the cover of annual herbs presents a U-shaped (concave-up parabolic) variation trend, and decreases significantly at altitudes above 1100 m. Semi-shrub cover and shrub cover follow a similar pattern, both increasing first and then decreasing with altitude. Altitude has a marked effect on semi-shrub density, with highly significant decreases observed at 400 m and 1200 m. Perennial herb density and annual herb density show a consistent pattern, peaking at 1100 m and then decreasing very significantly above that elevation. In contrast, semi-shrub density exhibits little variation. Shrub biomass shows a clear decreasing trend with increasing altitude, and the same trend is observed for perennial herb. The concentration of peak coverage and density near 700–1100 m is descriptively consistent with a mid-elevation optimum, which could arise from either mid-domain geometric constraints or environmental filtering.

**Fig 8:**
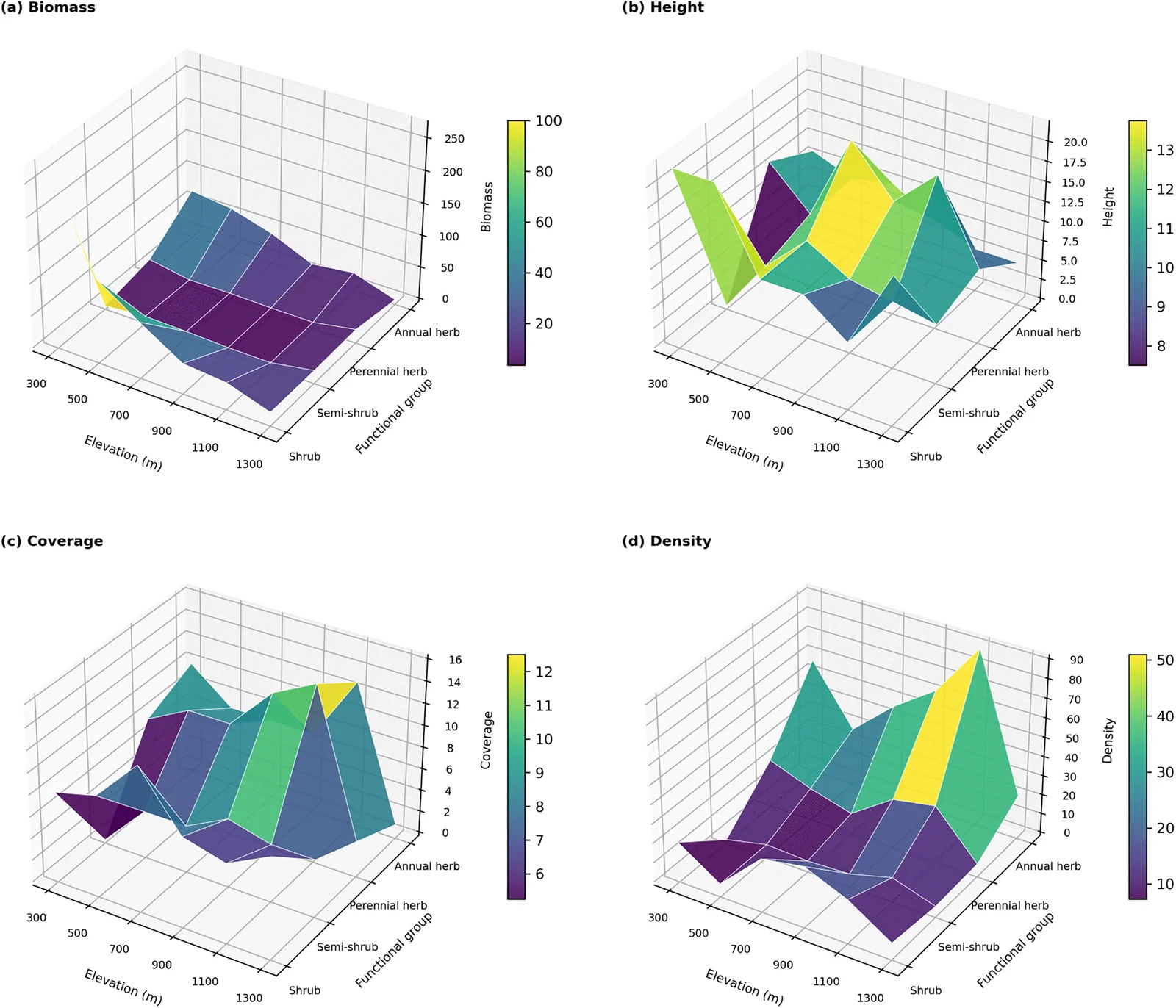
Spatial variation of plant eco-economic groups and elevation in Tacheng Region.

Collectively, these patterns demonstrate the structuring role of the elevational gradient in shaping desert plant communities in Northern Xinjiang. At low elevations, communities are dominated by shrubs and semi-shrubs, characterized by high aboveground biomass, tall plant stature, low population density, and low vegetation coverage, with community structure sustained by the individual size advantage of woody plants. In contrast, high‑elevation(1100m) communities are dominated by perennial and annual herbs, exhibiting high density, high coverage, low biomass, and short stature; herbaceous species adapt to high‑elevation environments via a numerical proliferation strategy. A pronounced turnover in community traits occurs along the elevational gradient: as elevation increases, the dominant ecological strategy shifts from a shrub‑driven resource‑acquisitive type to a herb‑driven numerical‑dominance type. This transition is ultimately the outcome of environmental filtering on plant life‑history strategies, driven by elevational changes in hydrothermal regimes and environmental stress intensity.

#### Community characteristics and elevational distribution in the Ili Desert.

As shown in Fig 9, the heights of shrubs, subshrubs and perennial herbs exhibited consistent variation trends along the elevation gradient. All three of these metrics reached their minimum values at elevations exceeding 1200 m, with each showing a significant decrease relative to lower elevations. In contrast, the height of annual herbs remained relatively stable across elevations. Shrub coverage increased significantly with rising altitude and presented a pronounced upward trend above 900 m. More specifically, the total vegetation coverage at 1200 m was higher than the corresponding observation value obtained at 200 m.Subshrub coverage displayed an initial decrease followed by a subsequent increase, while annual herb coverage conformed to a unimodal distribution pattern. Shrub density showed a remarkable rising tendency as elevation increased. The densities of subshrubs, perennial herbs and annual herbs all followed an upward-then-downward trajectory, and annual herb density experienced evident fluctuating variations. The biomass of shrubs, subshrubs and annual herbs generally declined with increasing elevation.

**Fig 9:**
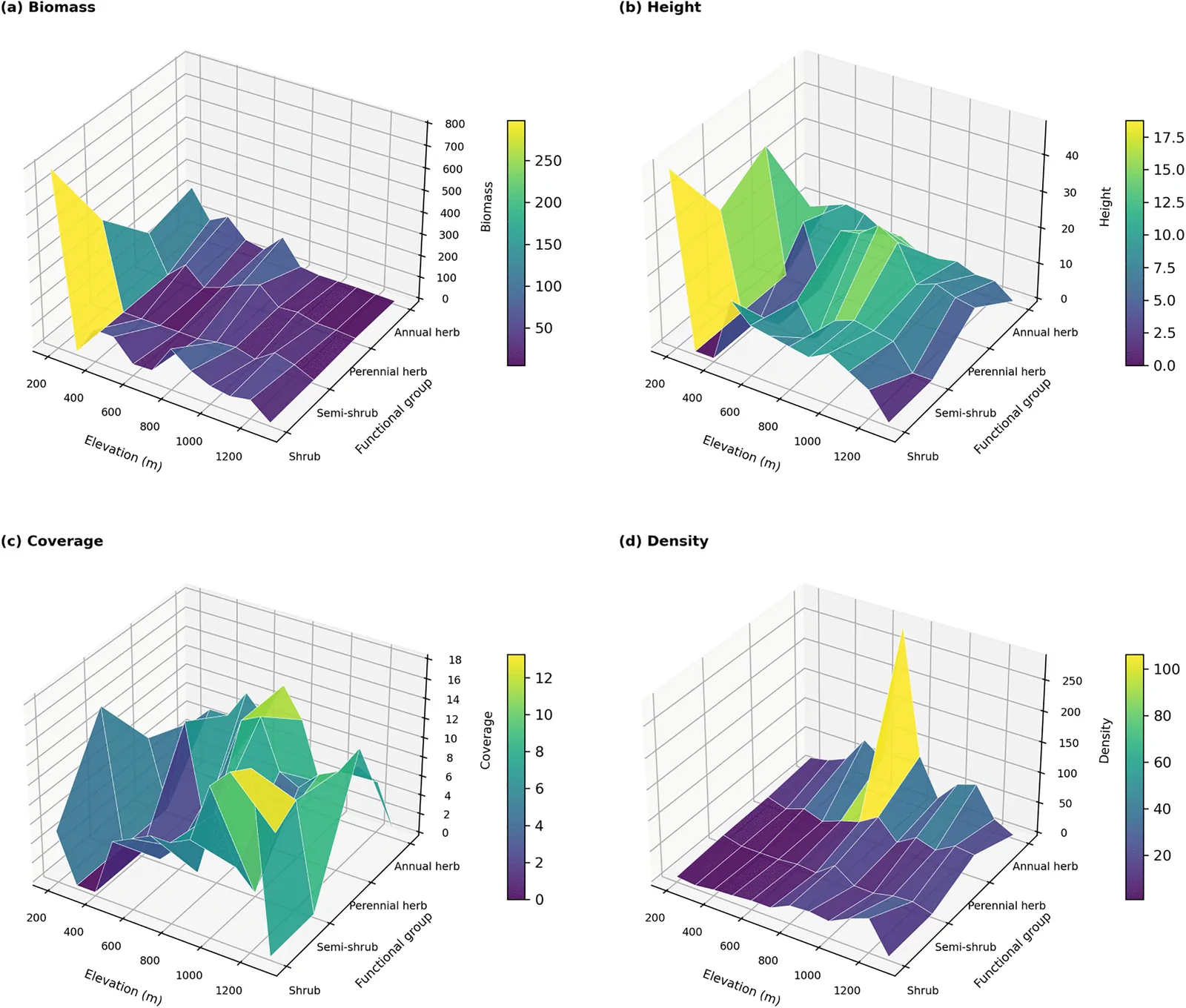
Spatial variation of plant eco-economic groups and elevation in Yili Region.

#### Community characteristics and latitudinal distribution in the Ili Desert.

As presented in Fig 10, shrub height, perennial herb height and annual herb height exhibited consistent variation trends along the latitudinal gradient, which increased first and then decreased. Conversely, subshrub height declined initially and then ascended. The coverage of subshrubs and annual herbs showed an increasing tendency. Compared with the corresponding coverage values observed at 43°N, the coverage of the two vegetation types at 45°N both increased to varying degrees. Shrub and perennial herb coverage decreased gradually with latitudinal change. Subshrub density increased steadily as latitude elevated. Annual herb density experienced a significant trend of initial reduction followed by elevation. No distinct variations were detected in the density of shrubs and perennial herbs. The maximum shrub biomass appeared at 44°N, while subshrub biomass reached its peak value at 45°N.

**Fig 10:**
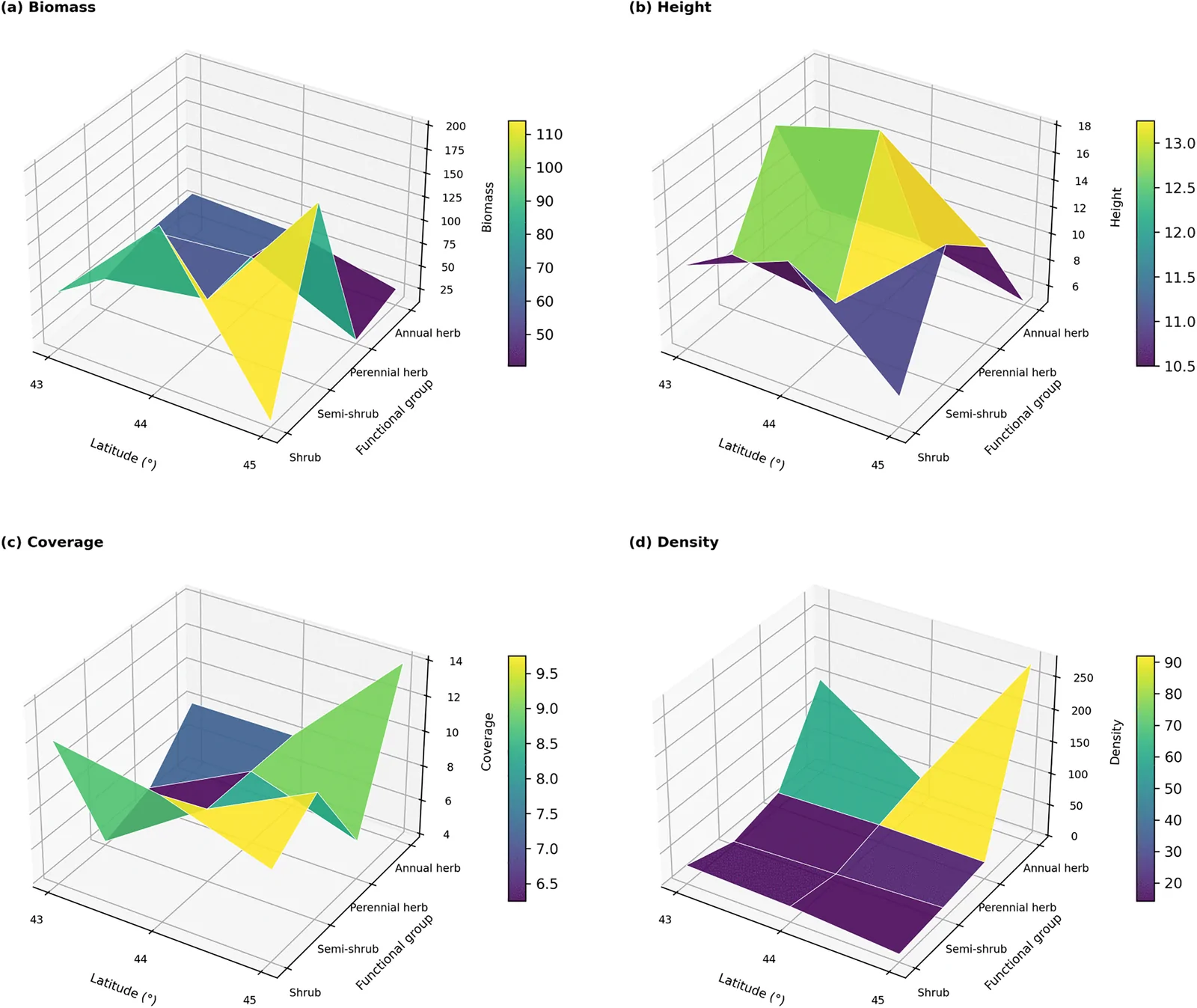
Spatial Variation of plant eco-economic groups and latitude in Yili Region.

#### Community characteristics and longitudinal distribution in the Ili Desert.

As shown in Fig 11, the heights of four plant life forms generally conformed to a unimodal trend along the longitudinal gradient. At 84°E, the height of shrubs decreased significantly, while the height of subshrubs exhibited an increasing trend.. No substantial variations were observed in the heights of perennial and annual herbs. The coverage of subshrubs, perennial herbs and annual herbs decreased first and then increased slightly with longitudinal change, with a relatively narrow variation range. Shrub density exhibited a similar dynamic to annual herb density, both rising initially and falling thereafter. Subshrub and perennial herb densities presented a notable decreasing trend, and annual herb density declined markedly at 83°E and 84°E. The biomass of shrubs, subshrubs and annual herbs attained the maximum at 83°E, followed by a continuous decreasing trend as longitude varied.

**Fig 11:**
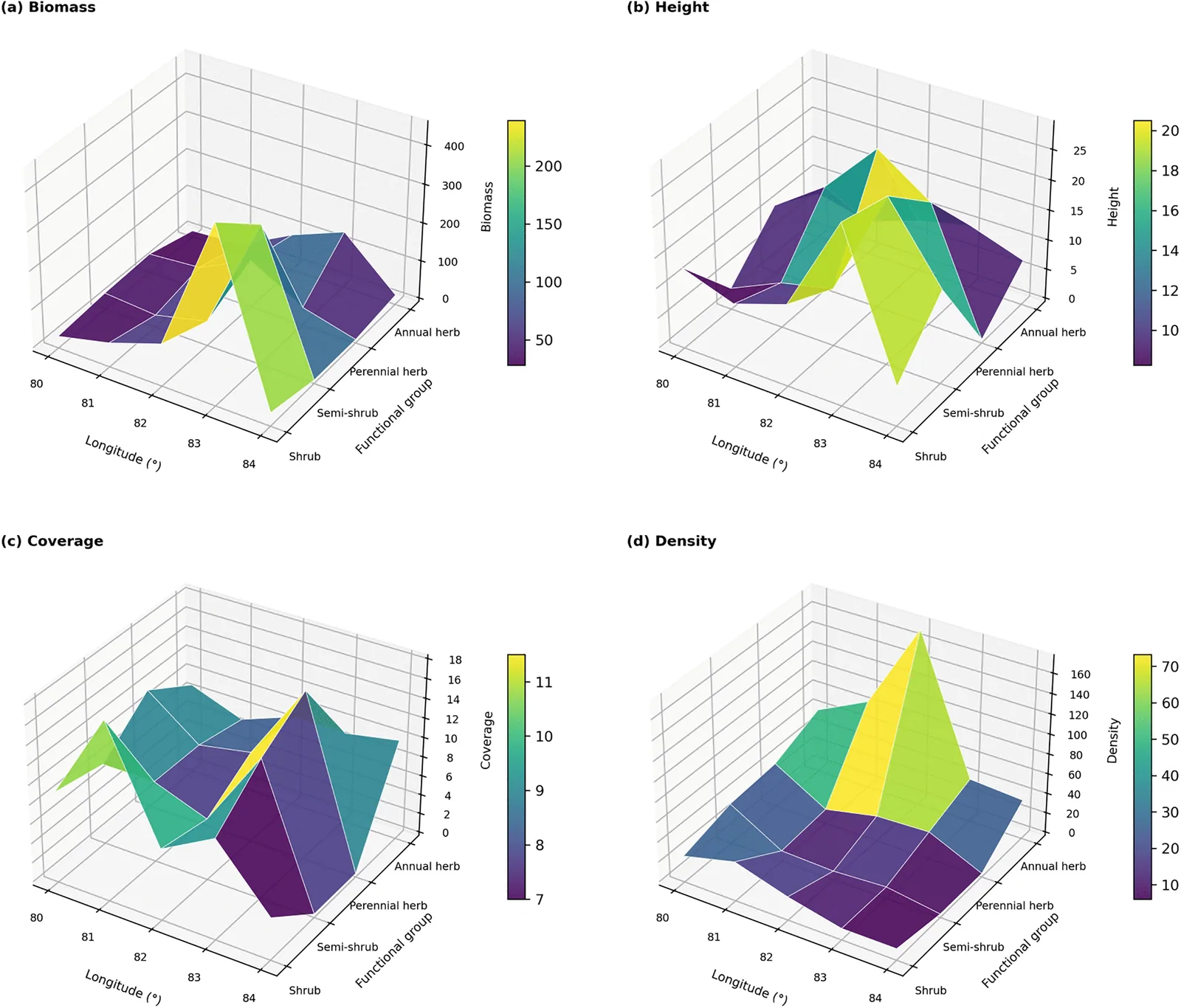
Spatial variation of plant eco-economic groups and longitude in Yili Region.

### Spatial patterns in the Bortala desert region

#### Altitudinal patterns in the Bortala desert.

As shown in Fig 12, the height of four plant life forms in the Bortala area presented an overall declining trend along the elevation gradient. Minor increases in shrub height and perennial herb height were detected at 500 m and 700 m respectively. Compared with the elevation range of 300–500 m, the heights of shrubs and perennial herbs at 1100 m decreased.The coverage of shrubs, perennial herbs and annual herbs exhibited a significant trend of initial increase and subsequent decrease with elevation variation. Shrub, perennial herb and annual herb density all followed an upward-then-downward pattern. Shrub and perennial herb density reached the maximum at 900 m, and annual herb density at 800 m was markedly higher than that at other altitudes. Shrub biomass peaked at 900 m and gradually declined with further elevation rise. Annual herb biomass attained its maximum value at 500 m, followed by a continuous decreasing trend as altitude increased. The unimodal coverage patterns peaking near 700–900 m are descriptively consistent with a mid-elevation optimum, while the overall declining height trend may reflect low-temperature limitation at high elevations. These patterns are consistent with both MDE and environmental filtering explanations.

**Fig 12:**
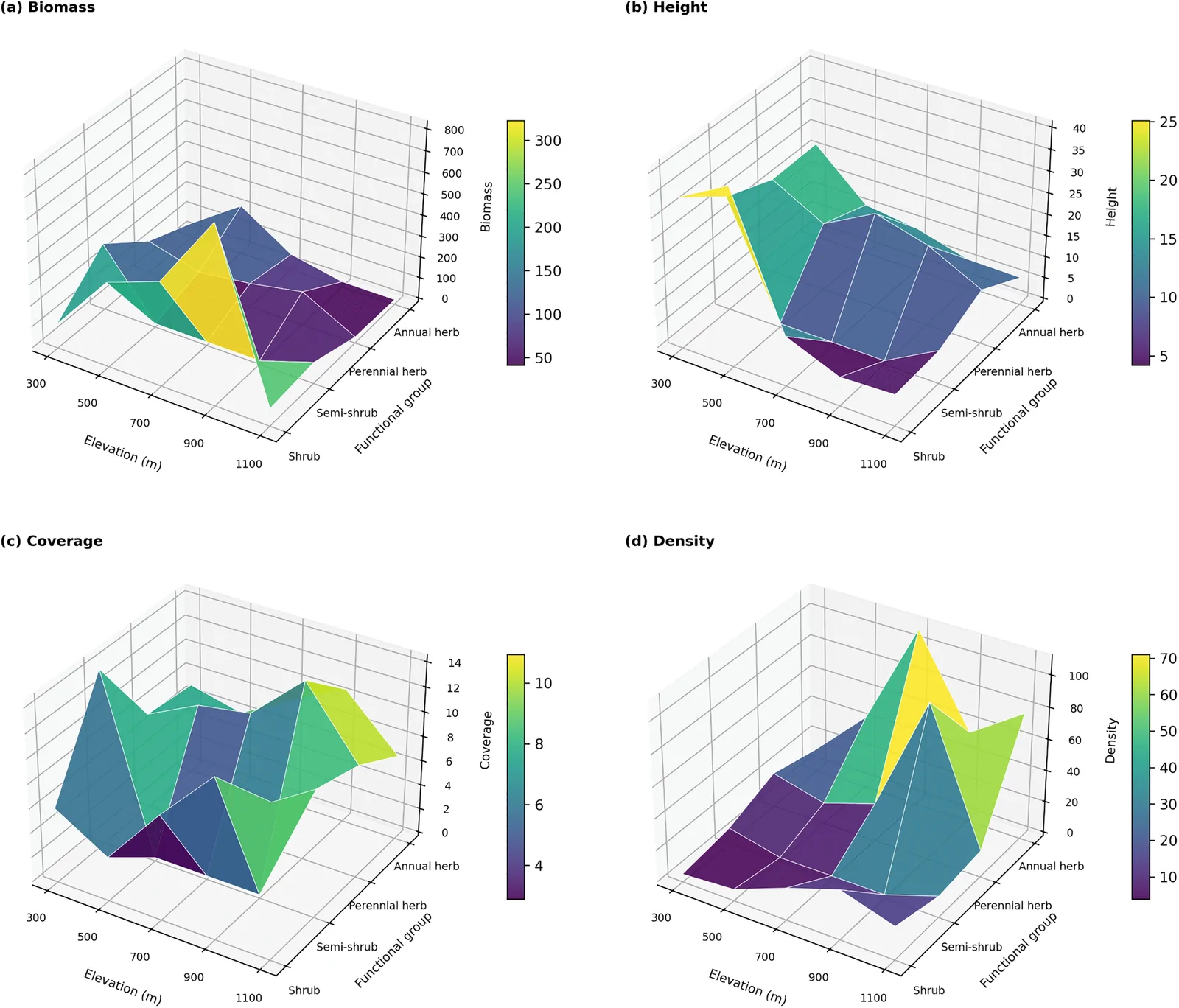
Spatial variation of plant eco-economic groups and elevation in Bortala Region.

#### Latitudinal patterns in the Bortala Desert.

As illustrated in Fig 13, the height of shrubs showed a decreasing trend along the latitudinal gradient, and a significant reduction was detected at 45°N. Conversely, the height of annual herbs exhibited a significant unimodal pattern of variation along the gradient, which first increased and then decreased, with the maximum value observed at 44°N, representing increase relative to other latitudes.Latitudinal variation only caused significant changes in shrub coverage, and a substantial reduction in shrub coverage was detected at 45°N. Shrub density and annual herb density exhibited divergent variation patterns across the latitudinal gradient. Specifically, compared with the 43°N latitude, shrub density at 45°N decreased significantly, while the density of annual herbs at 45°N exhibited a marked increase. Notably, the biomass of shrubs, subshrubs, perennial herbs, and annual herbs all reached their maximum values at the latitude of 44°N.

**Fig 13:**
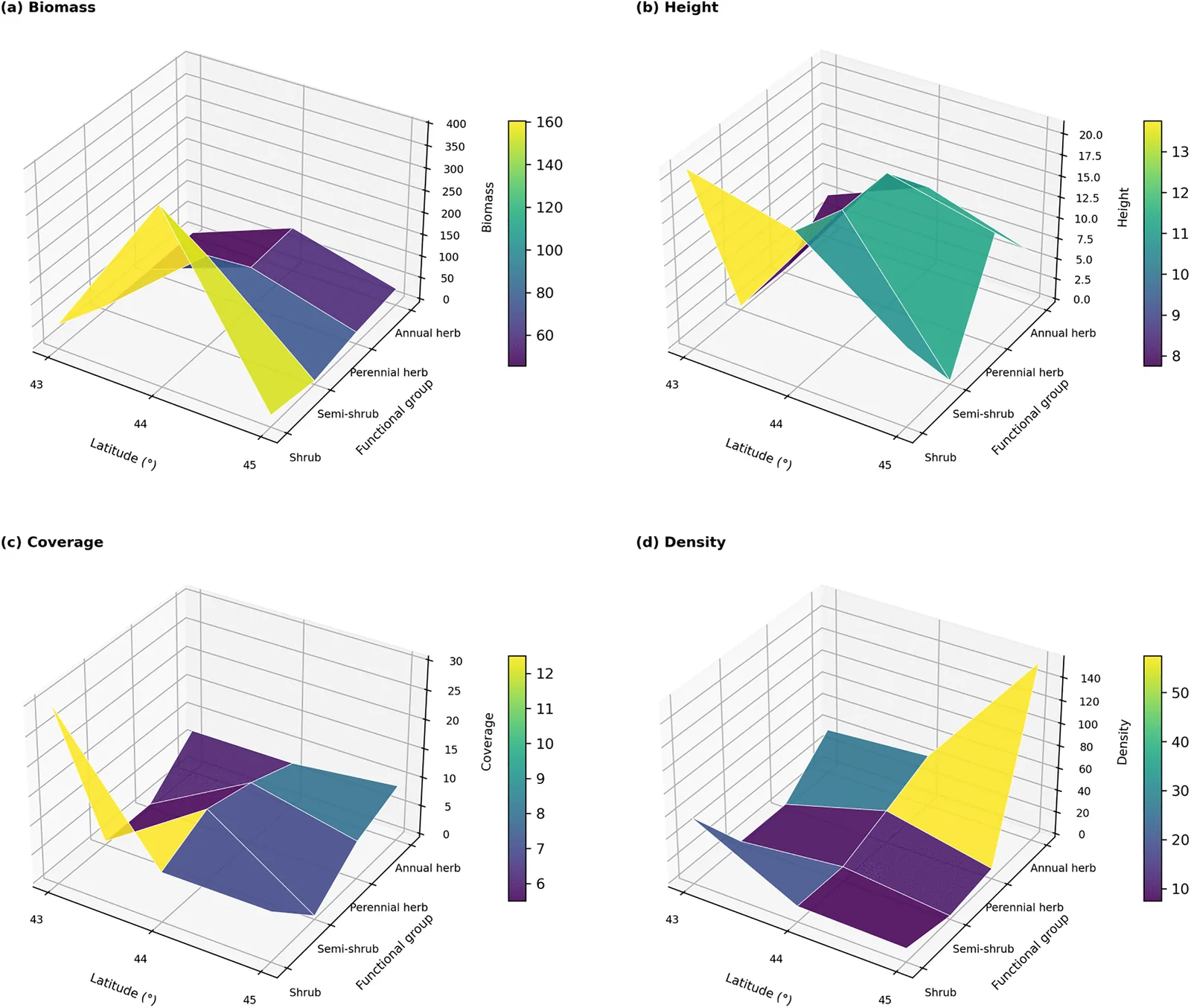
Spatial distribution of desert plant ecological economic groups and latitudes in the Bortala Region.

#### Longitudinal patterns in the Bortala Desert.

As illustrated in Fig 14, shrub and annual herb heights exhibited analogous significant increasing trends along the longitudinal gradient, with a slight drop detected at 84°E. Perennial herb height rose first and then fell, hitting the minimum value at the same longitude. Perennial herb coverage presented a gradual declining tendency, whereas annual herb coverage increased steadily with longitudinal shift.The density of annual herbs showed an initial decreasing trend followed by a subsequent increasing trend, reaching its maximum at 85°E, which was significantly higher than the corresponding value observed at 81°E. Perennial herb density decreased continuously across the longitudinal gradient. Shrub biomass maximized at 84°E. Subshrub and annual herb biomass attained their peak values at 83°E, and both declined gradually with growing longitude.

**Fig 14:**
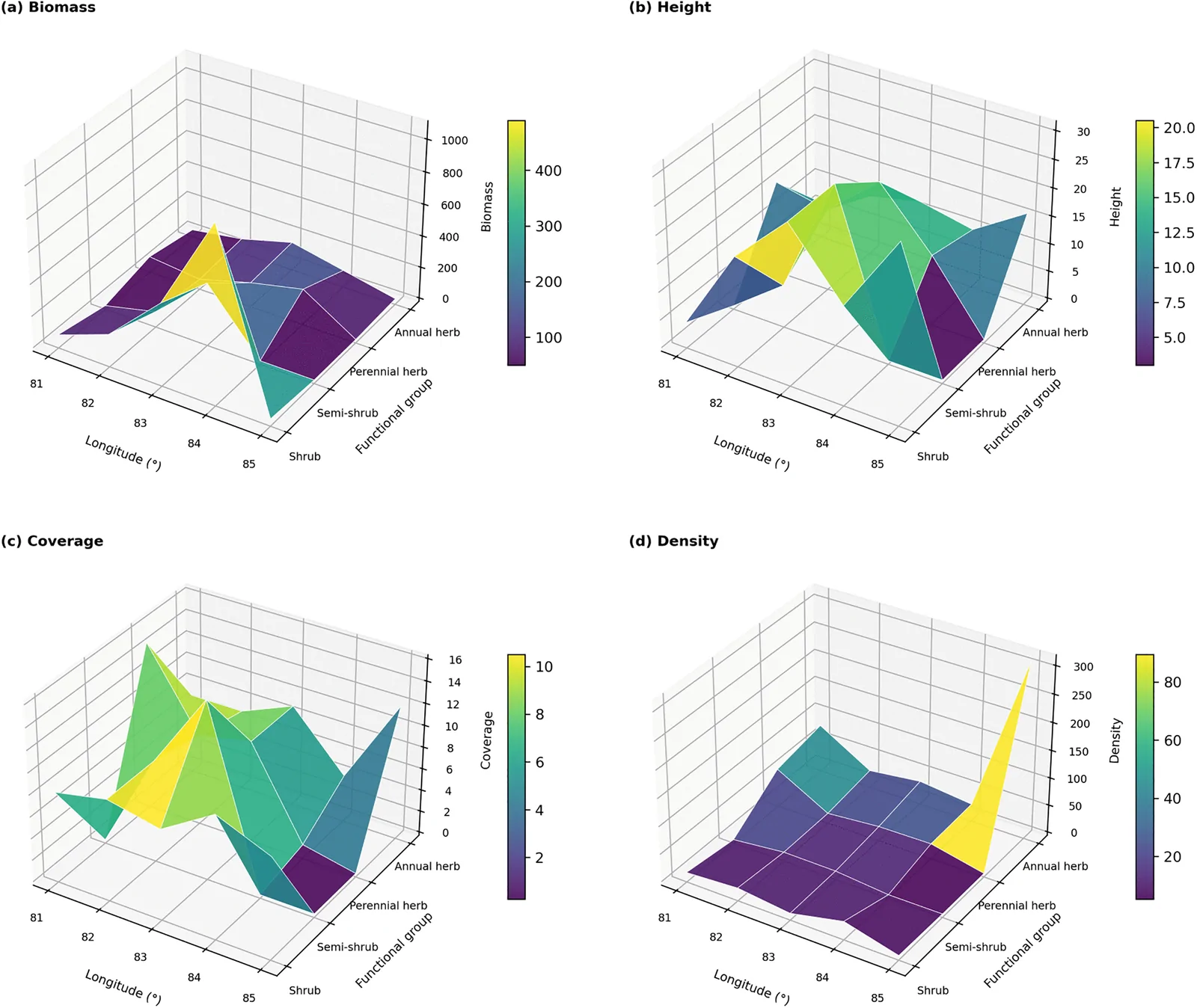
Spatial variation of plant eco-economic groups and longitude in Bortala Region.

### Spatial patterns in the Changji–Urumqi desert region

#### Altitudinal patterns in the Changji–Urumqi Desert.

As indicated in Fig 15, the maximum heights of subshrubs, perennial and annual herbs were all detected at the elevation of 700–800 m. The peak height of shrubs emerged at 500 m and 1200 m. Maximum coverage of all plant life forms concentrated in the range of 700–1000 m, while the minimum coverage across the community occurred at 1200 m. Shrub and annual herb densities remained relatively high and varied substantially from 600 m to 1000 m. Perennial herb density declined progressively along the elevation gradient, and subshrub density showed insignificant fluctuations. Collectively, the biomass of shrubs, subshrubs and perennial herbs displayed a declining tendency with rising elevation. The concentration of maximum heights and coverage within 700–1000 m is descriptively consistent with a mid-elevation optimum; this pattern is consistent with the MDE expectation but cannot be distinguished from environmental filtering without a geometric null model.

**Fig 15:**
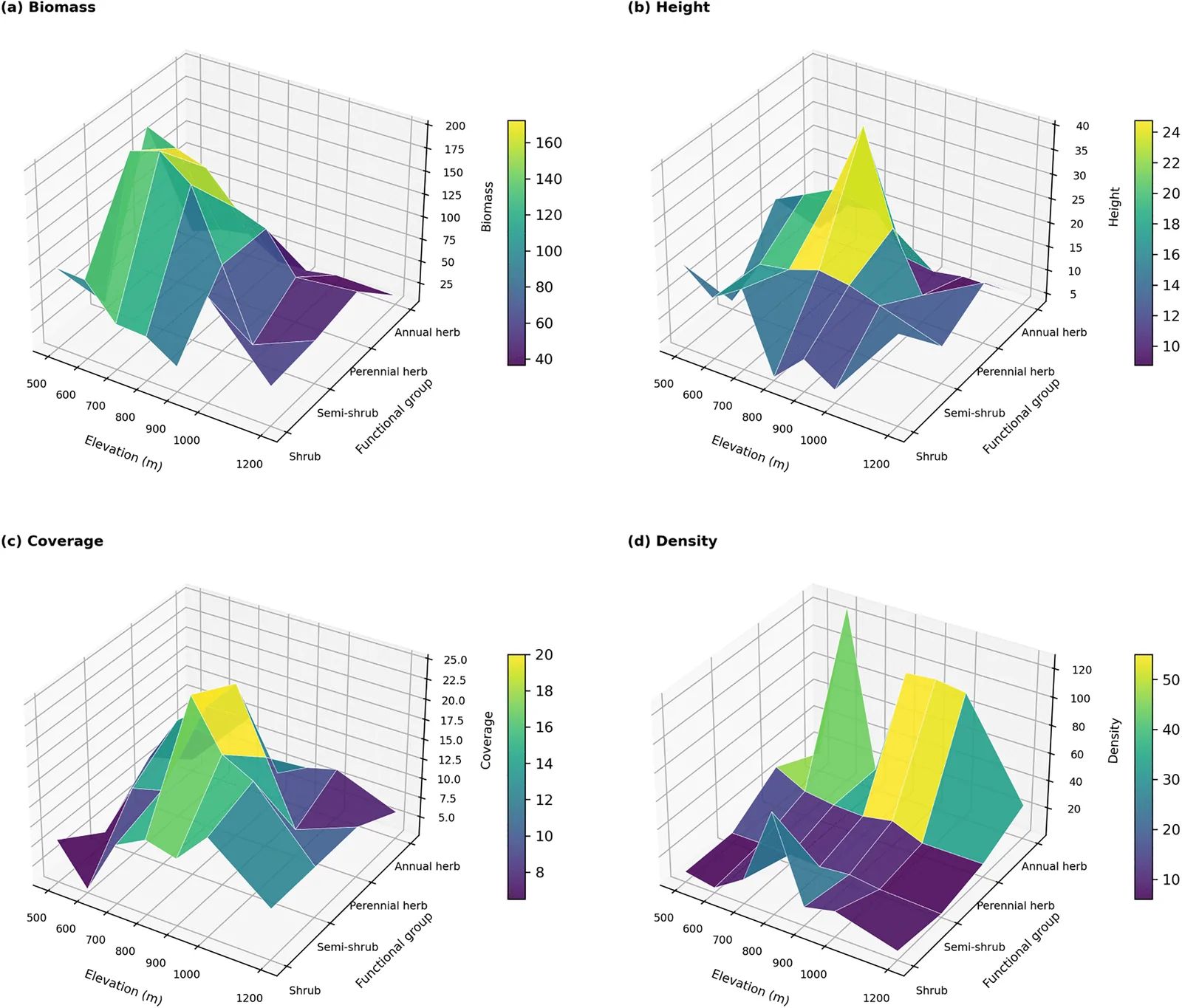
Spatial variation of plant eco-economic groups and elevation in Changji-Urumqi Desert.

#### Latitude patterns in the Changji–Urumqi Desert.

As illustrated in Fig 16, the heights of shrubs, subshrubs and perennial herbs followed an increasing-then-decreasing pattern along the latitudinal gradient. At 45°N, the heights of shrubs and subshrubs decreased significantly, and the height of perennial herbs also showed a notable reduction. By contrast, annual herb height increased gradually yet showed no substantial variation. The coverage of shrubs, subshrubs, perennial herbs and annual herbs was all negatively correlated with latitude. Shrub, subshrub and annual herb densities declined significantly with increasing latitude, while no significant variation was observed in perennial herb density. Overall, the biomass of shrubs, perennial herbs and annual herbs presented a decreasing trend as latitude rose.

**Fig 16:**
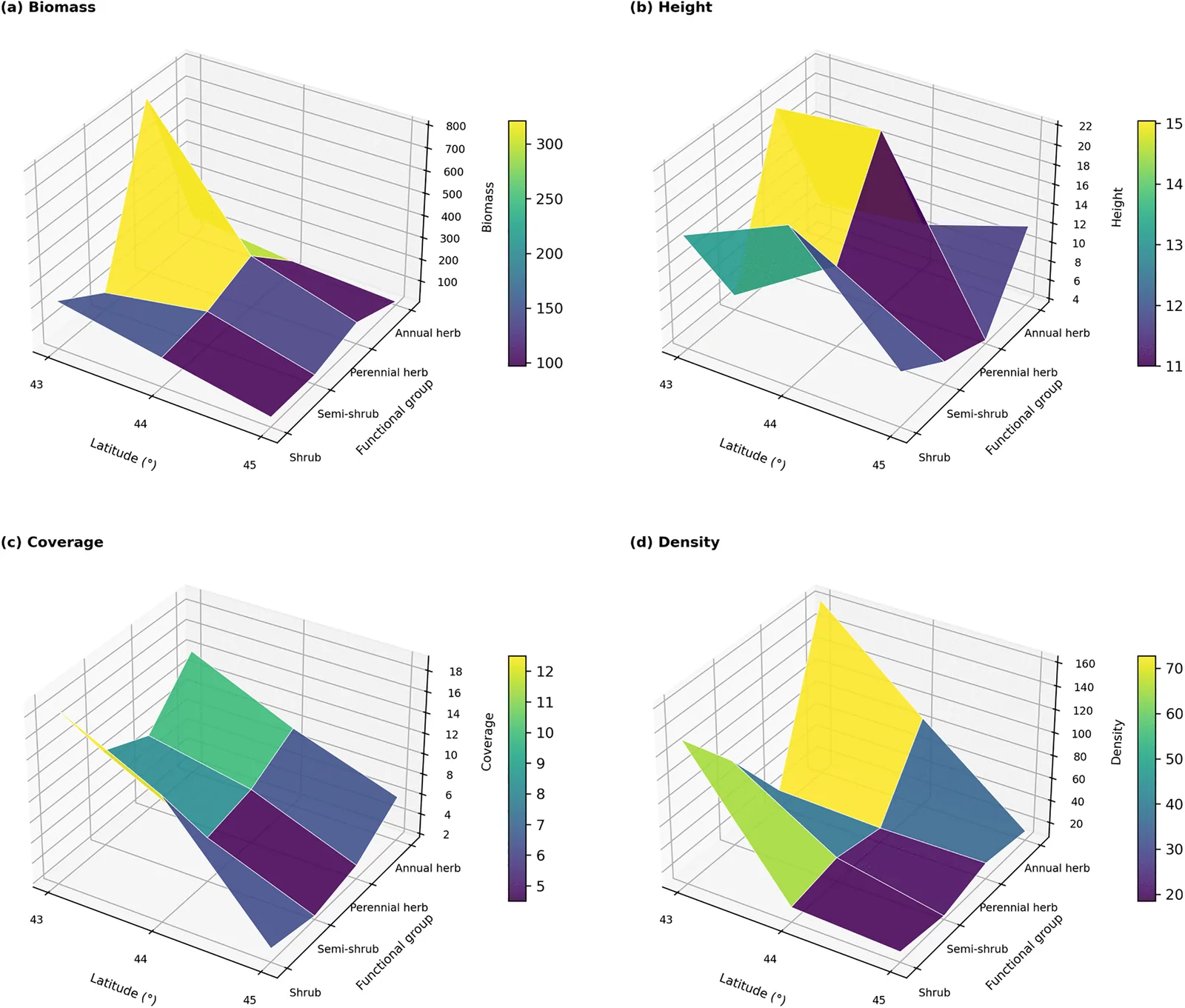
Spatial variation of plant eco-economic groups and latitude in Changji-Urumqi Desert.

#### Longitudinal patterns in the Changji–Urumqi Desert.

As shown in Fig 17, shrub, subshrub and annual herb heights generally declined along the longitudinal gradient. Perennial herb height followed a hump-shaped trend and reached its maximum at 87E. Shrub and subshrub coverage increased initially and then decreased, both peaking at 87E, while perennial herb coverage declined progressively with longitude. The minimum values of all four plant life forms were recorded at 90E. Shrub, subshrub and annual herb densities exhibited a significant increasing-then-decreasing pattern. The maximum densities of shrubs and subshrubs occurred at 87E, and that of annual herbs at 89E. No distinct trend was detected for perennial herb density. Total biomass increased steadily as longitude increased, with pronounced variations observed in shrubs and perennial herbs. The eastward decline in coverage toward 90E is descriptively consistent with a longitudinal gradient that may correspond to increasing aridity, though direct drought measurements were not available.

**Fig 17:**
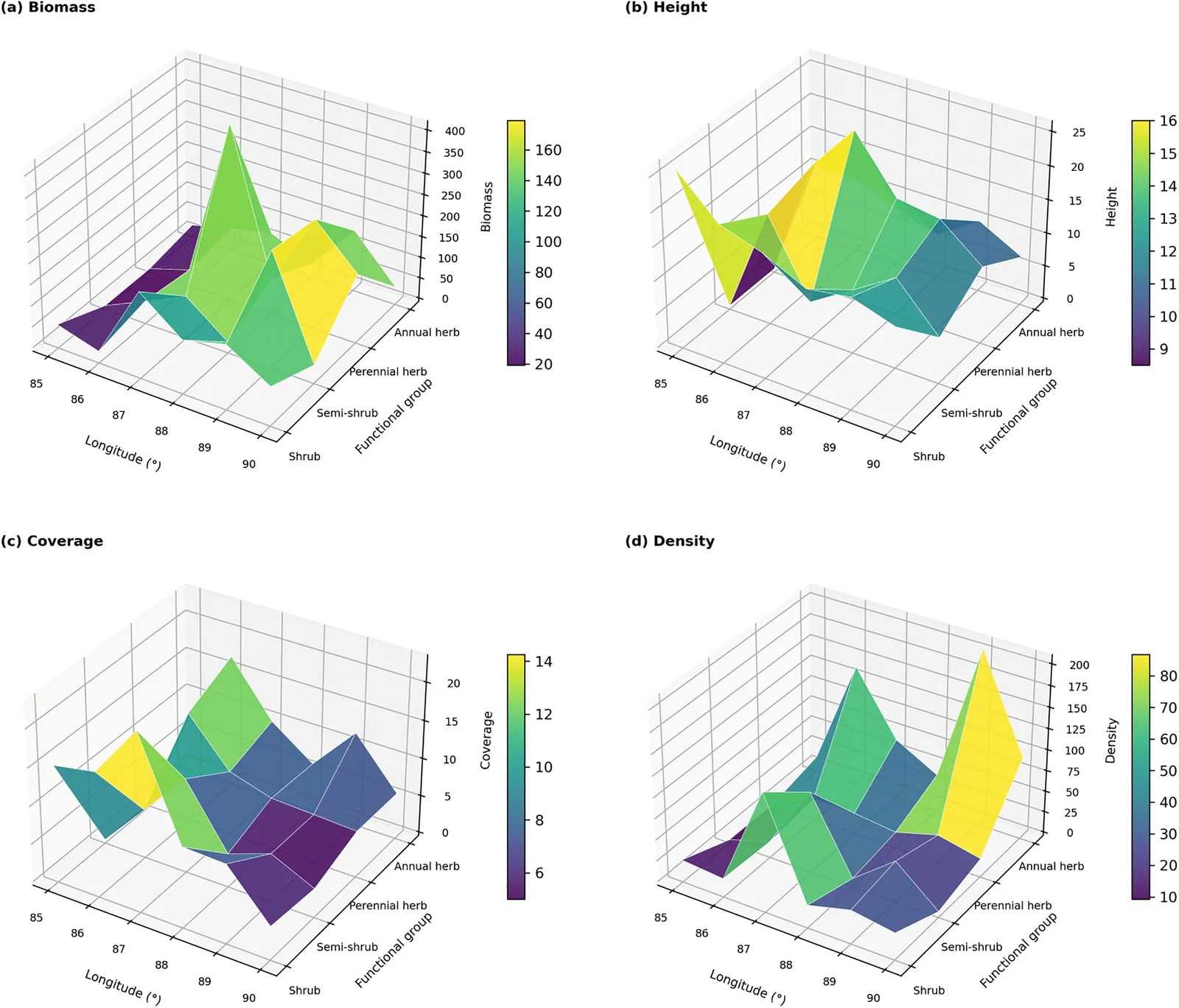
Spatial variation of plant eco-economic groups and longitude in Changji-Urumqi Desert.

### Spatial patterns in the East Tianshan Mountains desert region

#### Altitudinal patterns in the East Tianshan Desert.

As presented in Fig 18, along the elevation gradient, subshrub height decreased significantly at 1300 m relative to its maximum value, while annual herb height followed a hump-shaped pattern. Both perennial and annual herb coverage increased first and then decreased. Perennial herb coverage rose markedly at 1100 m, whereas subshrub coverage experienced an initial significant decline followed by a recovery. The maximum coverage of subshrubs, perennial herbs and annual herbs was all observed at 1100 m. Densities of perennial and annual herbs exhibited an increasing-then-decreasing trend, and shrub density increased continuously with rising elevation. The density of perennial herbs increased significantly at the elevation of 1100 m. Noticeable variations were detected in the biomass of shrubs and subshrubs, which generally declined as elevation increased. Despite the overall arid conditions, the unimodal coverage patterns peaking near 1100 m are descriptively consistent with a mid-elevation optimum, possibly shifted toward higher elevations due to extreme aridity at low-elevation zones.

**Fig 18:**
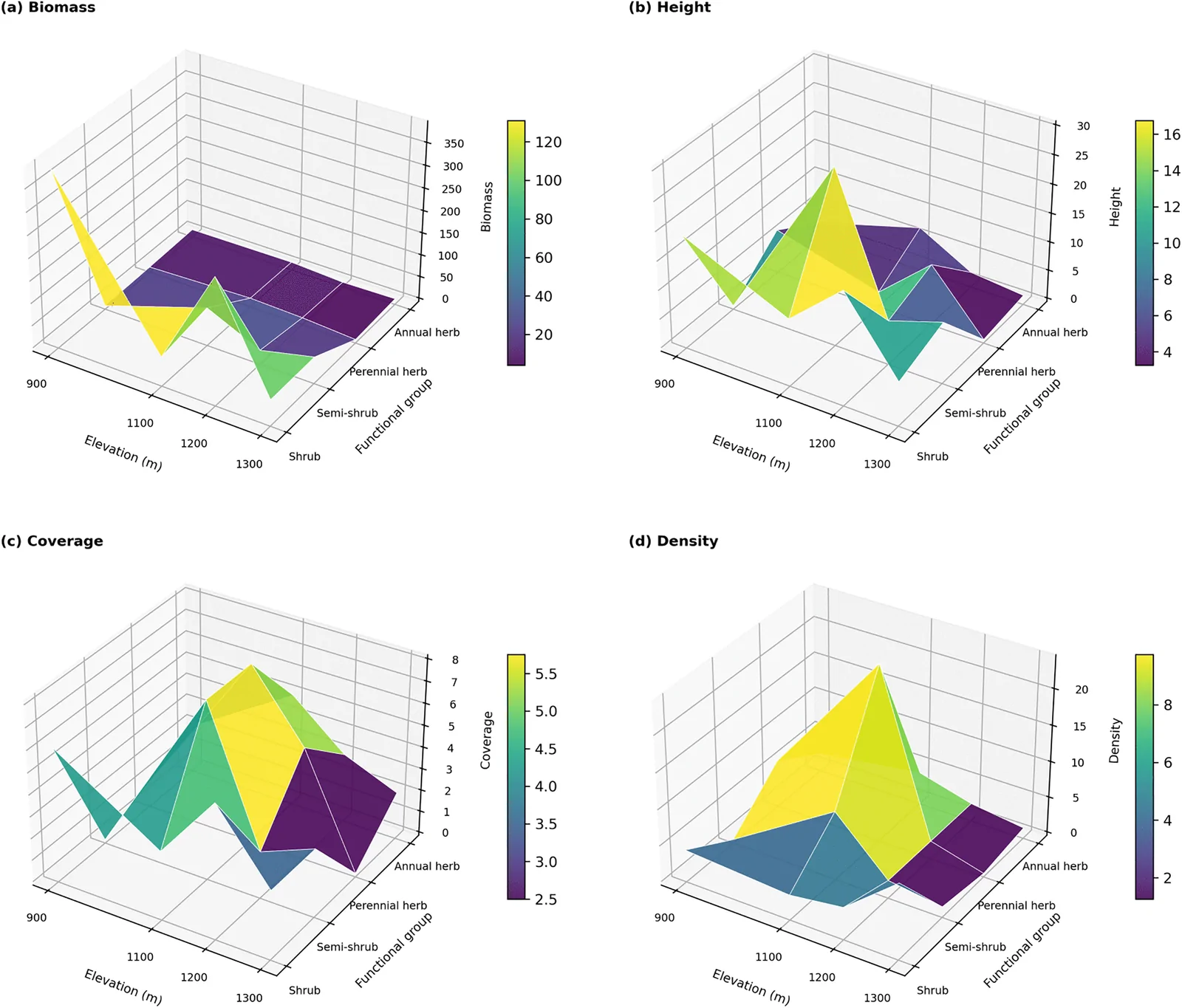
Spatial variation of plant eco-economic groups and elevation in the eastern of Xinjiang Desert.

#### Latitudinal patterns in the East Tianshan Desert.

As illustrated in Fig 19, shrub height and annual herb height shared similar hump-shaped trends along the latitudinal gradient. At the latitude of 44°N, shrub height increased, while at 45°N, subshrub height decreased significantly. Annual herb height showed no substantial variation, and perennial herb height displayed a trend of initial decrease followed by a rise. Shrub, perennial herb and annual herb coverage exhibited consistent increasing-then-decreasing patterns. The maximum coverage values of all four plant life forms were recorded uniformly at 44°N. The densities of both perennial herbs and annual herbs followed a hump-shaped distribution trajectory. The density of annual herbs increased at 44°N, then decreased sharply at 45°N. The biomass of shrubs and subshrubs peaked at 44°N and decreased gradually with further increases in latitude.

**Fig 19:**
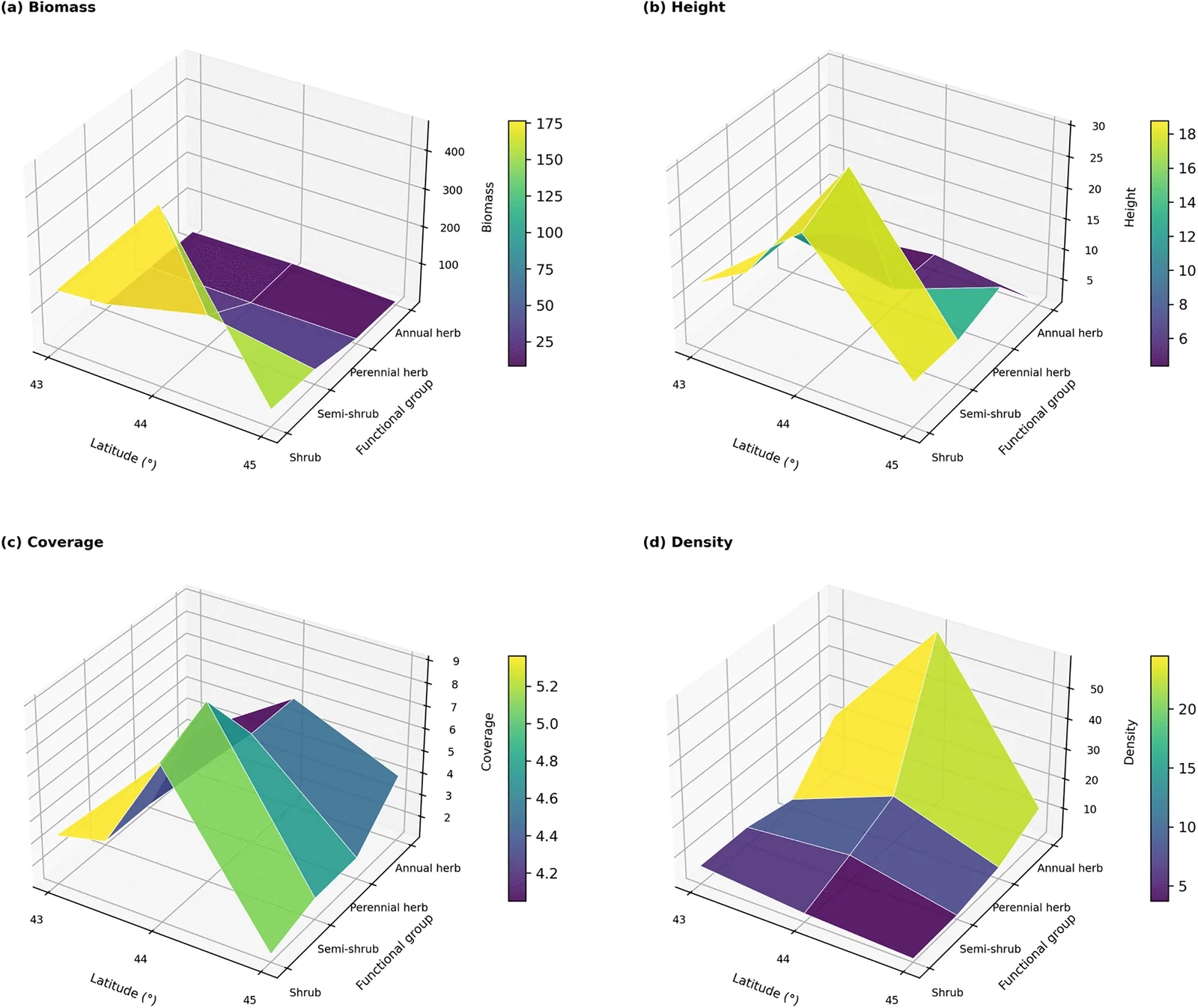
Spatial variation of plant eco-economic groups and latitude in the eastern of Xinjiang Desert.

#### Longitudinal patterns in the East Tianshan Desert.

As illustrated in Fig 20, subshrub height exhibited a significant decreasing trend along the longitudinal gradient, while perennial herb height followed a unimodal pattern of initial increase and subsequent decrease. In contrast, shrub height displayed an opposite trend, declining first and then increasing with longitudinal variation. Along the longitudinal gradient, the coverage of subshrubs and the height of perennial herbs showed a continuous decreasing tendency, whereas shrub height underwent no significant longitudinal variation. Densities of subshrubs and perennial herbs declined gradually with increasing longitude, with annual herb density presenting a particularly significant decreasing trend. Shrub biomass reached its maximum at 88E, while subshrub biomass peaked at 90E. The biomass of annual herbs decreased steadily along the longitudinal gradient, and no distinct regular trend was observed in perennial herb biomass. The divergent biomass responses of different plant life forms to longitudinal gradients reflect heterogeneous structural patterns across the eastern Tianshan region. The overall low community structural values within this subregion are descriptively consistent with the longitudinal gradient, which may correspond to the higher aridity in the easternmost part of the study area. However, direct measurements of soil moisture content or drought intensity were not acquired in this research.

**Fig 20:**
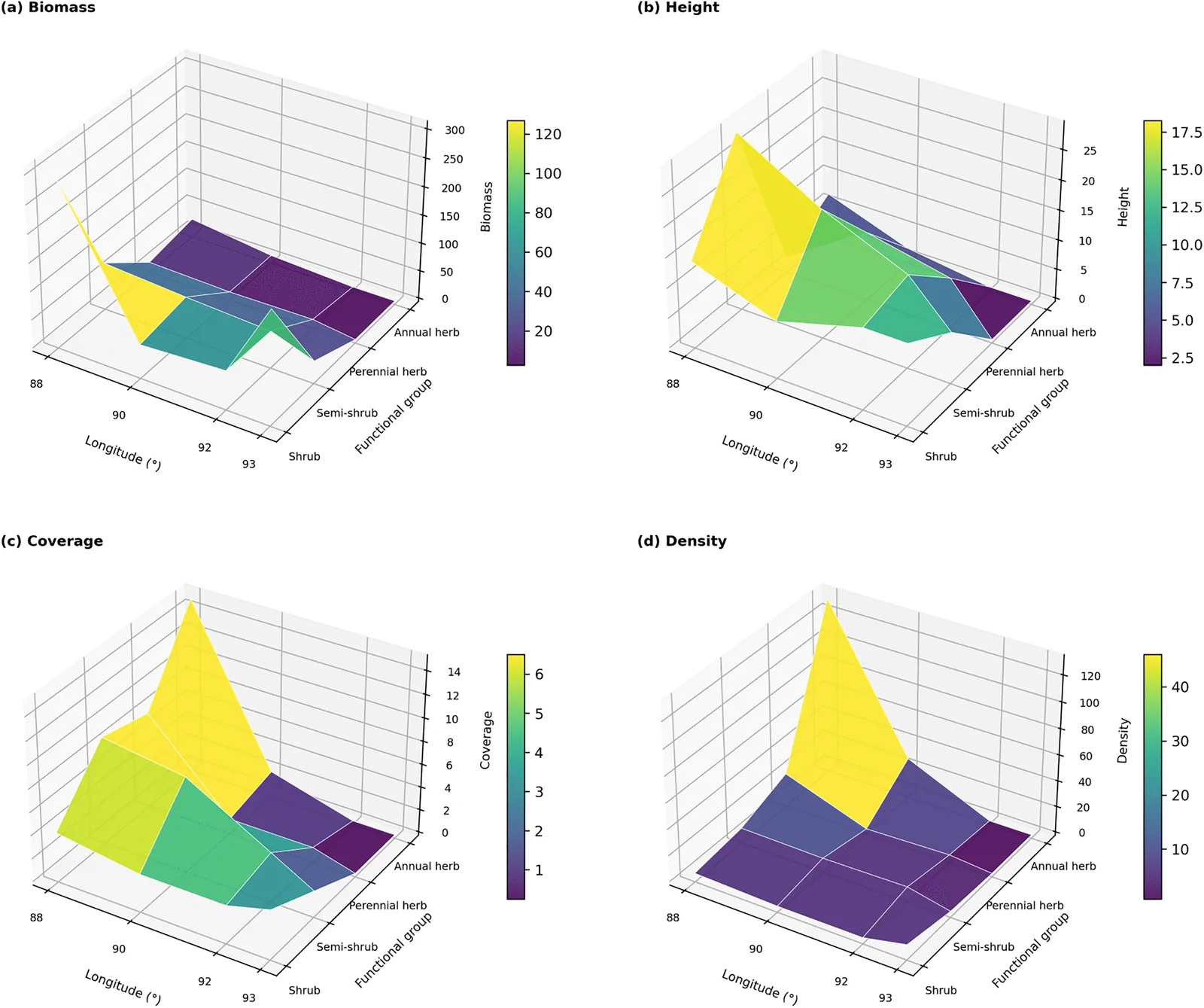
Spatial variation of plant eco-economic groups and longitude in the eastern of Xinjiang Desert.

Overall, the variations in community height, cover and density across the desert grasslands of northern Xinjiang are predominantly shaped by elevation. Community height declines consistently with increasing elevation, whereas community cover exhibits a unimodal pattern (increasing first and then decreasing) along the elevational gradient. Regarding community density, annual herbs follow a similar unimodal trend with elevation, while shrubs, semi-shrubs and perennial herbs show no significant elevational variation.Along the longitudinal gradient, community cover fluctuates markedly, whereas community height remains relatively stable with a narrow fluctuation range. The density of shrubs, semi-shrubs and perennial herbs increases initially, then decreases and levels off within the longitude band of 86°–88°, with a reduced magnitude of fluctuation. These patterns indicate that along the longitudinal transect, community cover and density in the Tacheng-Yili, Changji-Urumqi and Altay desert sub-regions are higher than those in the eastern Tianshan desert region; no significant difference in community height is detected across the sub-regions.Along the latitudinal gradient, shrub cover and the density of both annual herbs and shrubs show pronounced fluctuations, while all other plant life forms display no significant latitudinal trends. This further confirms that from north to south (i.e., from the Altay desert region southward to the Changji-Urumqi, Tacheng-Yili and eastern Tianshan desert regions), grassland community height follows a trajectory of initial increase followed by general stabilization. With the exception of shrub cover, total community cover across the sub-regions exhibits an overall unimodal trend (initial increase followed by subsequent decline) from north to south. For community density, no significant difference is observed among the desert sub-regions, with the exception of shrubs and annual herbs.

### Single-gradient pattern analysis of desert communities in Northern Xinjiang

As illustrated in Figs 21–29, Response pattern diagram of the quantitative characteristics of desert vegetation communities in six representative study areas of northern Xinjiang along an environmental gradient.three types of spatial environmental gradients are involved: the elevation gradient (Elevation, m), which reflects the combined variation of hydrothermal conditions along the vertical direction; the latitude gradient (Latitude, °N), which reflects the thermal difference across the north-south direction (higher values indicate more northerly latitudes and lower temperatures); and the longitude gradient (Longitude, °E), which reflects the moisture difference across the east-west direction (higher values indicate more easterly longitudes and stronger aridity).From left to right, the panels correspond to four quantitative characteristics of desert vegetation communities: height (Height, cm), coverage (Coverage, %), density (Density, ind./m^2^), and biomass (Biomass, g/m^2^).

**Fig 21:**
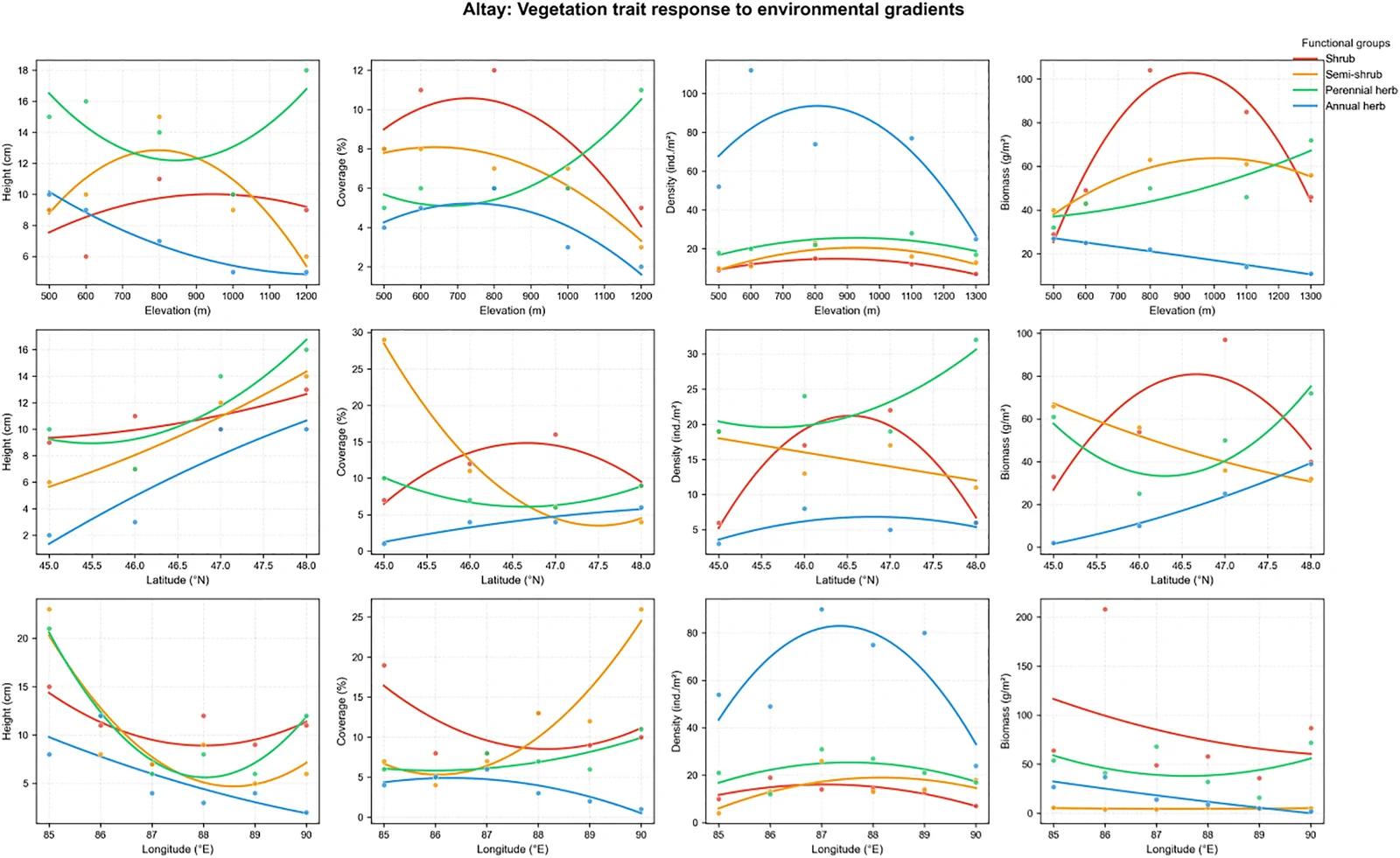
Altay vegetation trait response to spatial gradients.

**Fig 22:**
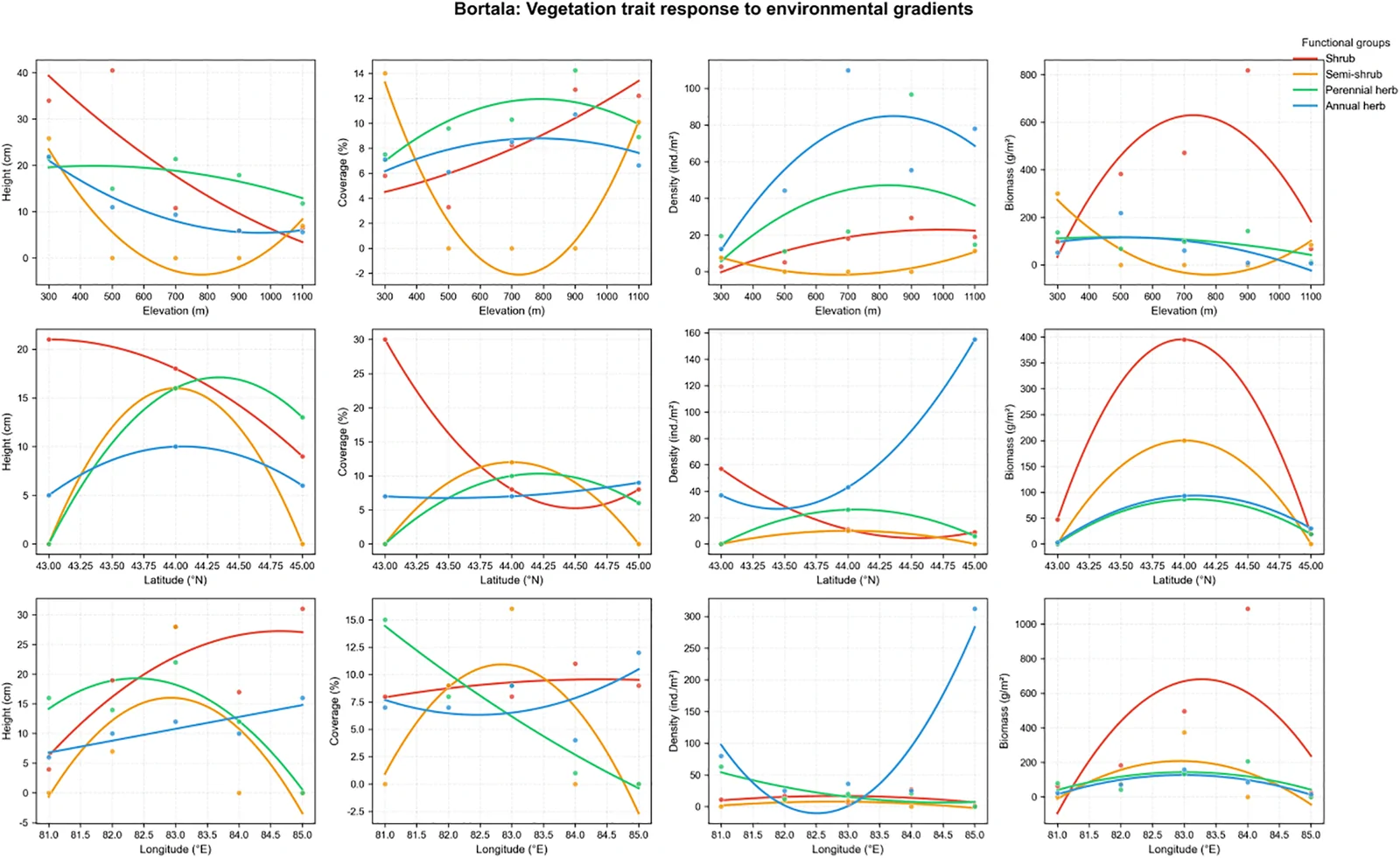
Bortala vegetation trait response to spatial gradients.

**Fig 23:**
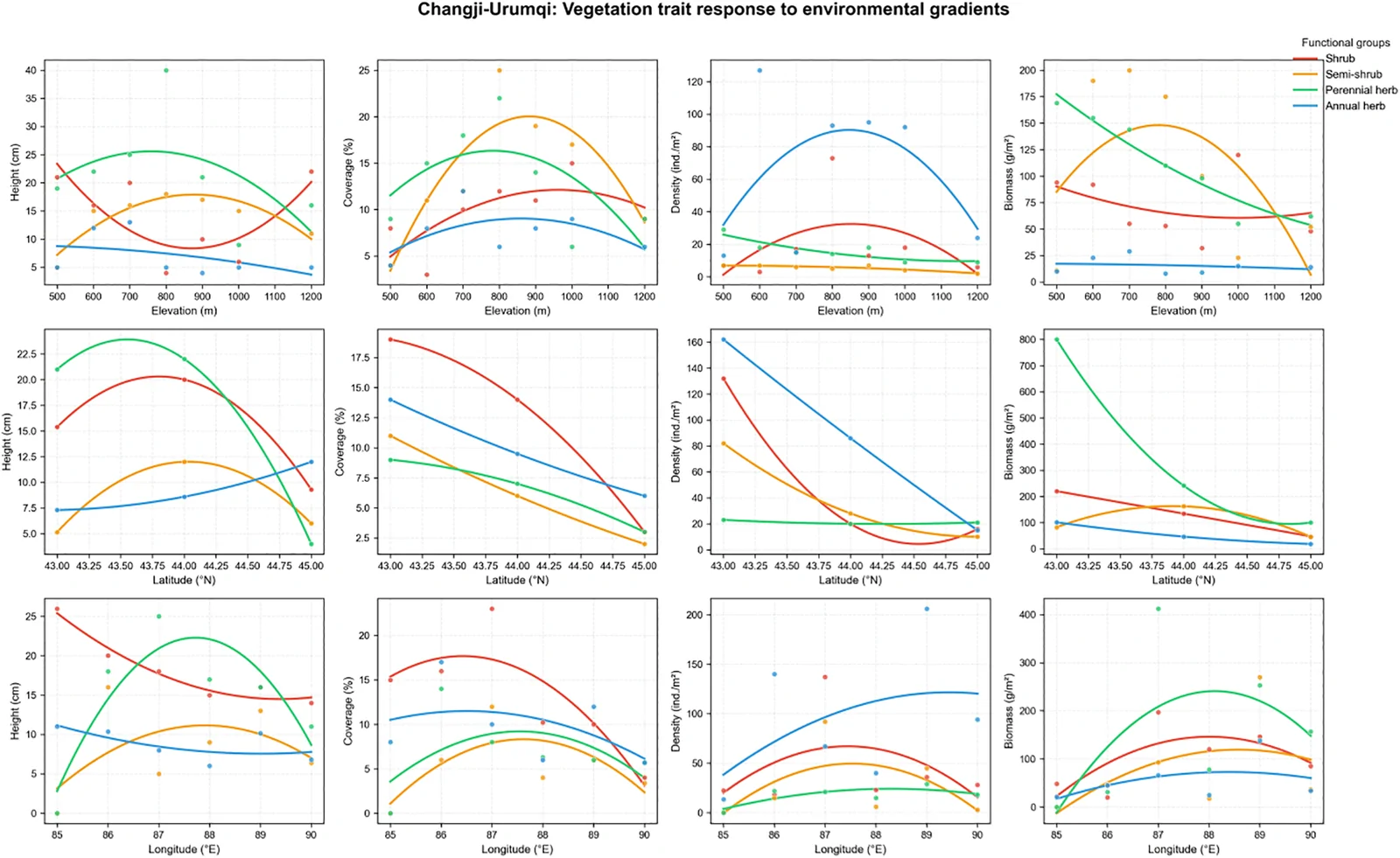
Changji-Urumqi vegetation trait response to spatial gradients.

**Fig 24:**
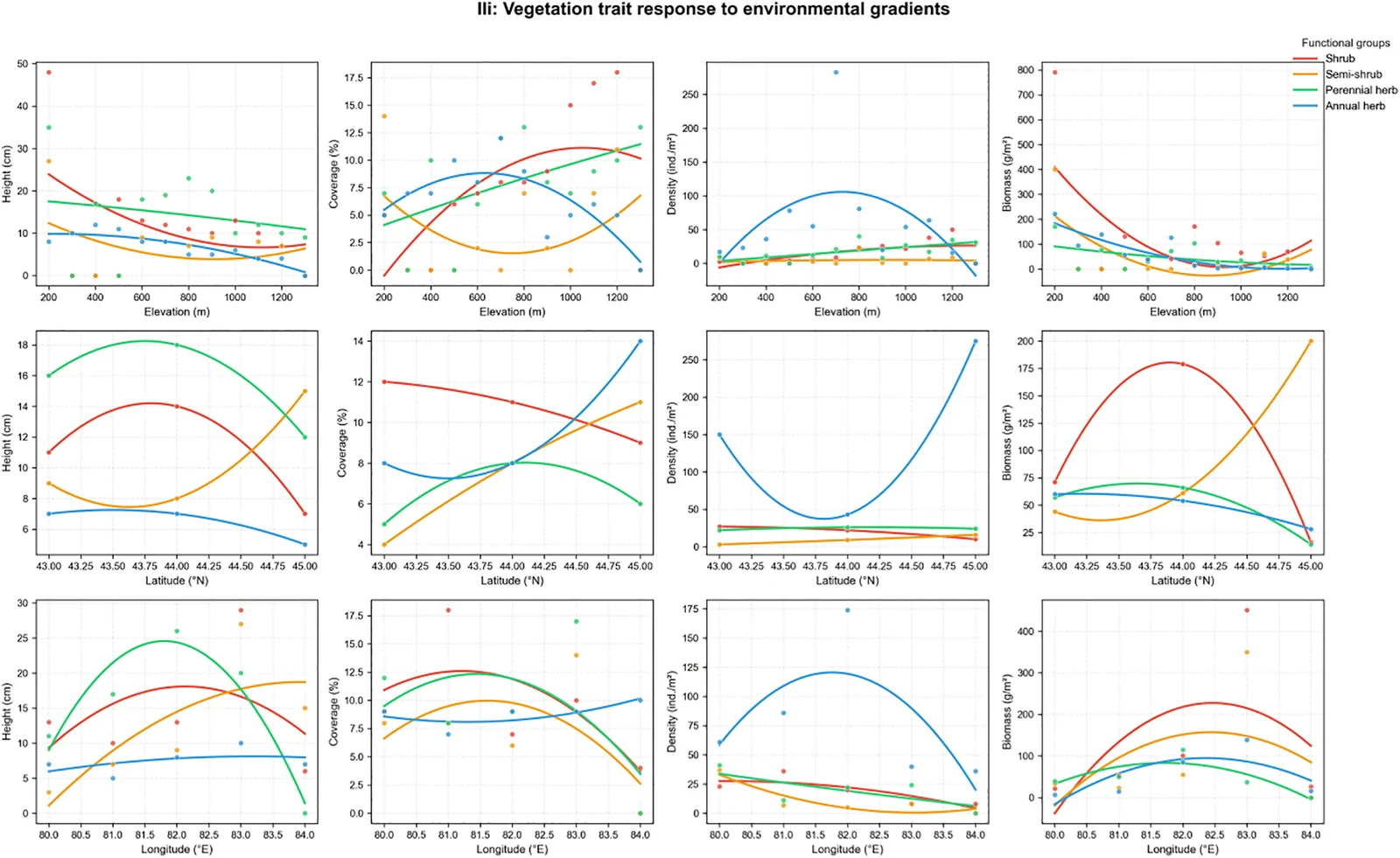
Lli vegetation trait response to spatial gradients.

**Fig 25:**
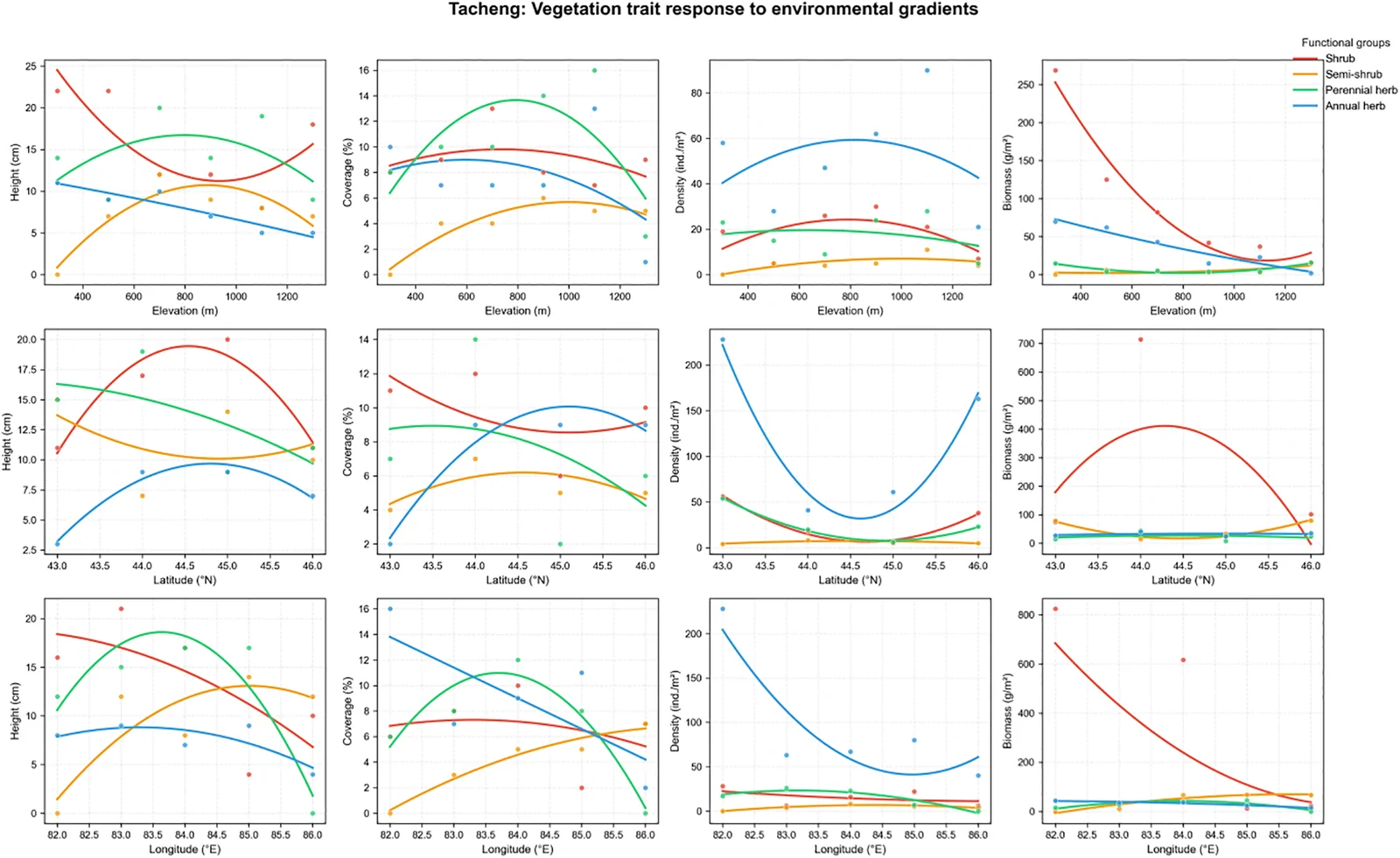
Tacheng vegetation trait response to spatial gradients.

**Fig 26:**
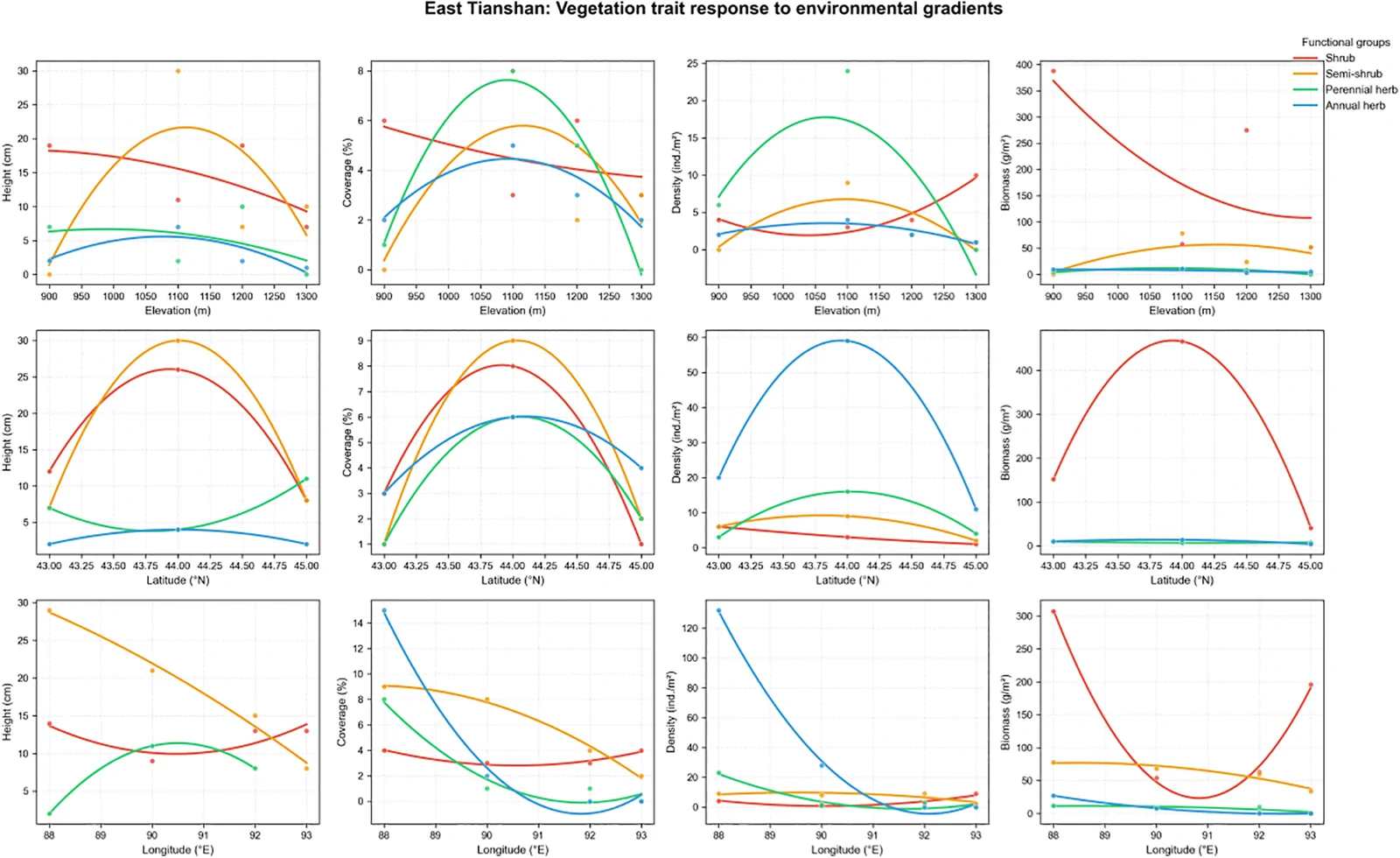
Eastern Tianshan mountains vegetation trait response to spatial gradients.

**Fig 27:**
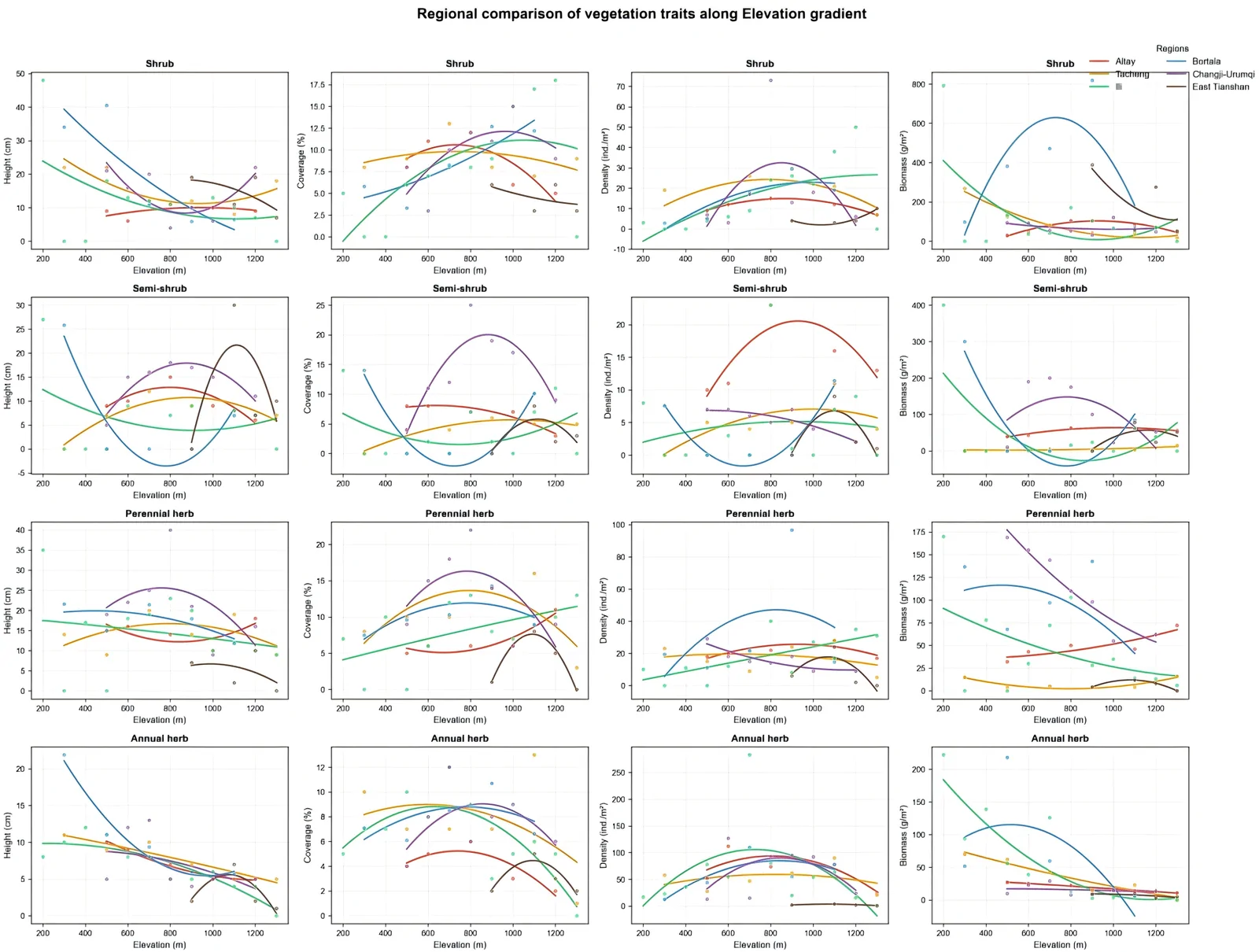
Elevation (cross-regional comparison curve).

**Fig 28:**
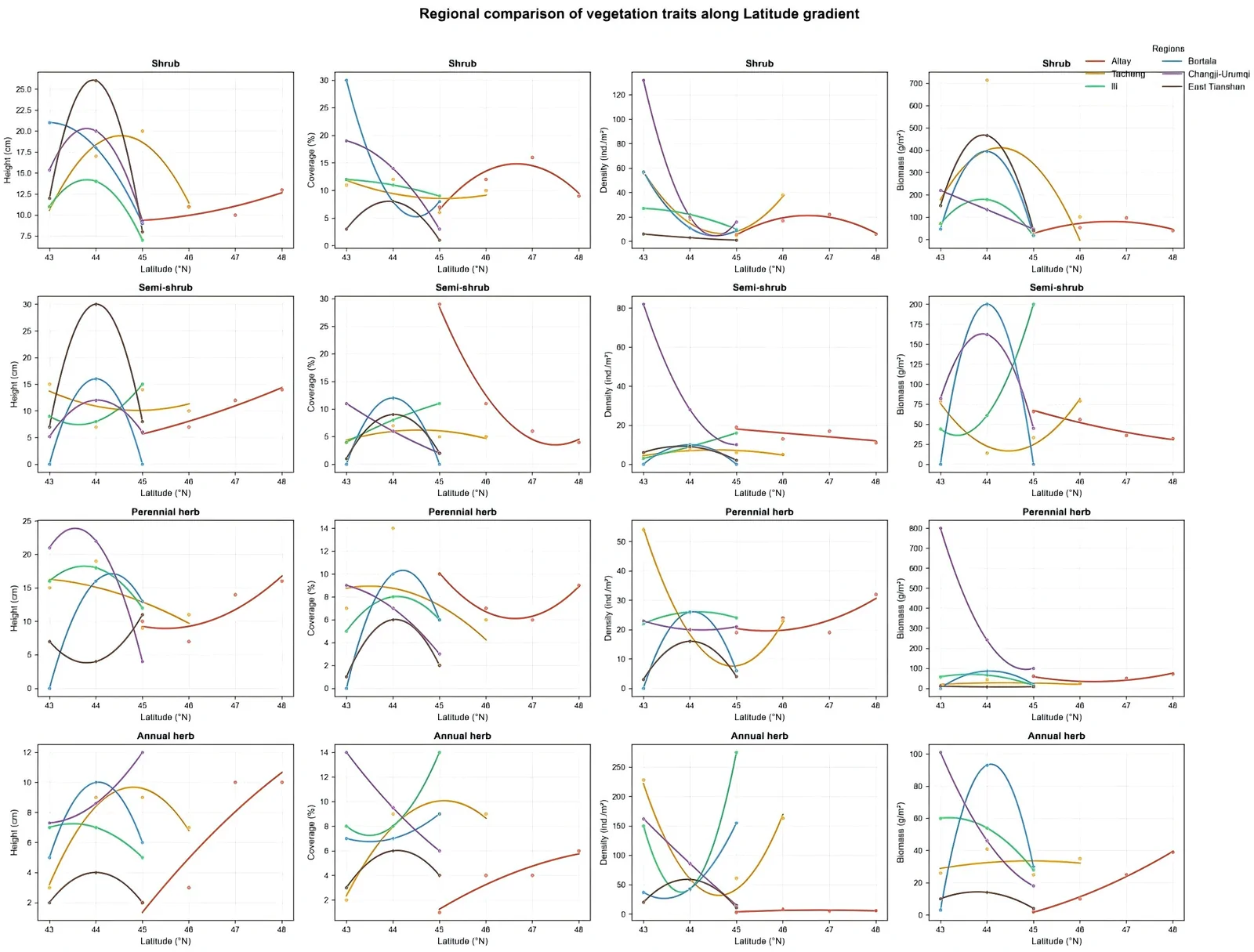
Latitude (cross-regional comparison curve).

**Fig 29:**
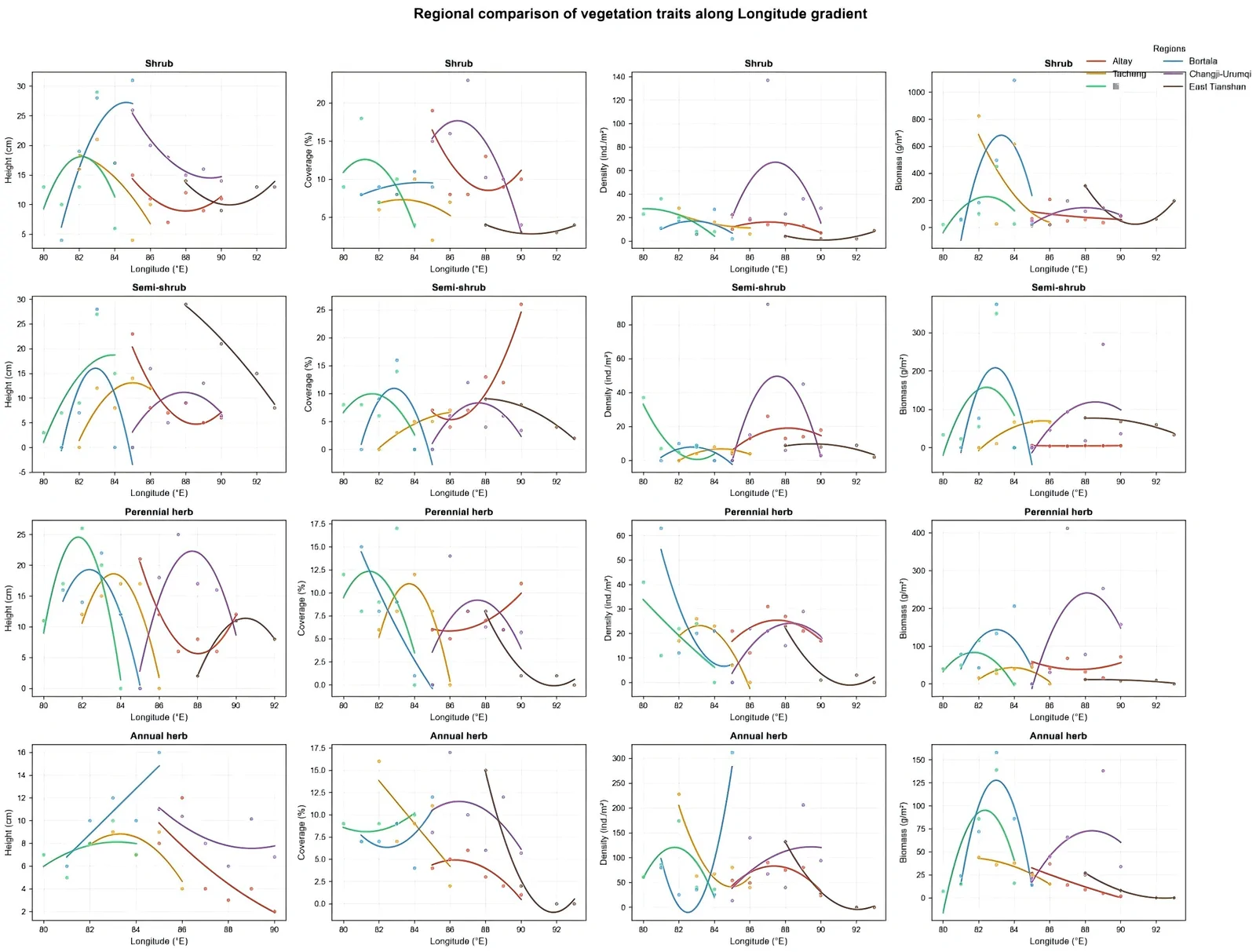
Longitude (cross-regional comparison curve).

As illustrated in Figs 21–29, the Altay desert region occupies the northernmost portion of the study area. Its mountainous zones receive relatively abundant precipitation, and vegetation exhibits clear vertical differentiation. The region features a complex geomorphological mosaic: its western sector acts as a corridor for moist cold air masses from the Atlantic Ocean, its eastern part is influenced by the arid Mongolian climate system, the southwestern section forms part of the western Junggar Mountains, and the south adjoins the Junggar Basin. In accordance with the official grassland regionalization framework of Xinjiang, this area is classified as the temperate desert mountain grassland region of northern Xinjiang.Across this desert region, the height and biomass of shrub and semi-shrub functional groups display a characteristic unimodal pattern, with peaks centered at the mid-elevation belt of 700–800 m where hydrothermal conditions are optimal. The cover and biomass of perennial herbs increase gradually with elevation, reflecting their strong adaptability to low-temperature environments. Annual herb density exhibits a pronounced unimodal trend, reaching a maximum at mid-elevations, whereas growth is constrained by low temperatures at higher elevations. Along the latitudinal gradient (northward), the height and density of herbaceous species generally increase, consistent with the facilitative effect of higher precipitation in northern areas. Shrub biomass follows a unimodal distribution, with the most favorable hydrothermal balance occurring at mid-latitudes. Along the longitudinal gradient from west to east, aridity intensifies progressively: shrub height, cover, and biomass decline continuously; annual herb density peaks in the central segment and decreases again in the east due to drought stress; and perennial herbs show a slight recovery in the eastern sector, where their drought tolerance confers a competitive advantage.

The Bortala desert grassland is situated between the Alataw and Borohoro Mountains in the western Tianshan range. Although surrounded by mountains on three sides that block cold moist westerly airflows, its westerly location and relatively low mountain relief allow the penetration of moist air masses. At low elevations (approximately 300–500 m), shrubs attain exceptional height and biomass, corresponding to the dominant habitat of valley shrub communities. Annual herb density exhibits a highly pronounced unimodal pattern, peaking at mid-elevations (ca. 600–700 m), a pattern driven by locally well-matched hydrothermal conditions. Semi-shrub cover displays a U-shaped response, with higher values at both low and high elevations and suppression at mid-elevations due to competition from herbaceous species. Given its relatively narrow latitudinal range, shrub communities decline overall with increasing latitude, whereas annual herb density increases in the northern sector, where local ephemeral habitats support explosive growth of annual plants. As aridity increases from west to east, shrubs continue to degrade, while annual herb density rises in the eastern section, reflecting the substitution effect of annual herb synusia in arid ecosystems.

The Changji–Urumqi grassland region is located on the northern slope of the Tianshan Mountains and in the southern Junggar Basin. Natural grassland resources in this region show prominent north–south differentiation, as well as west-to-east variation most evident in moisture availability, with conditions becoming progressively more arid eastward. These climatic and topographic conditions directly determine grassland types and vegetation functional traits. The complex topography exerts strong control over regional climate: the relative elevation difference between mountains and plains reaches 2000–4500 m in both western and eastern parts, generating pronounced vertical climatic zonation. Most plant functional groups exhibit a canonical unimodal response, with peaks concentrated in the 600–800 m piedmont oasis–mountain transition zone, consistent with the vertical zonation pattern of the northern Tianshan slope. Annual herb density reaches an exceptionally high peak, corresponding to the extremely well-developed annual herb synusia at oasis margins; perennial herb biomass attains high values at low elevations, sustained by irrigation water from oases. With increasing latitude (northward), the height, cover, and density of shrubs and semi-shrubs decrease overall, highlighting a thermal limitation effect; herbaceous groups also show varying degrees of decline. Along the longitudinal gradient, community quantitative traits generally follow a *high in the middle, low at both ends* pattern, with values declining at the eastern end due to aridity or topographic constraints.The Ili desert grassland, located in the western Tianshan Mountains, is surrounded by mountains to the north, east, and south and opens westward to form a U-shaped valley that intercepts water vapor from the westerlies. It is the region with the most favorable moisture regime and most vigorous vegetation development across the entire study area. At low elevations (200 m), shrubs in valley shrub communities have relatively high height and biomass, both of which decrease with increasing elevation. Perennial herbs have a broad ecological amplitude, with their height and cover peaking at mid-elevations; annual herb density follows a unimodal pattern, with optimal hydrothermal matching at mid-elevations. Overall vegetation cover and biomass are higher than those in eastern regions. With a narrow latitudinal range, shrub height decreases gradually with increasing latitude, while annual herb density increases with latitude, as local habitats in the north support the development of annual synusia. Along the longitudinal gradient from west to east (80°E to 84°E), shrub traits exhibit a unimodal trend; as aridity intensifies in the eastern sector, perennial herbs decline and the relative proportion of annual herbs increases.

The Tacheng region, in northwestern Xinjiang, has zonal vegetation dominated by shrubs and semi-shrubs. Shrub height and biomass decrease continuously with elevation, and reed swamps occur in localized depressions at low elevations. Perennial herb height follows a unimodal pattern, with optimal hydrothermal balance at mid-elevations; annual herb density shows a distinct unimodal pattern, peaking at mid-elevations. Shrub height and cover are unimodally distributed, with the most favorable hydrothermal combination at mid-latitudes (ca. 44°N); annual herb density increases continuously with latitude, sustained by higher precipitation in the north. The Eastern Tianshan Mountains, at the easternmost end of the study area, experience severe drought stress and support relatively sparse overall vegetation, representing a typical arid ecosystem. Semi-shrub height and cover show the most pronounced unimodal pattern, with the optimal zone at mid-elevations (ca. 1050 m); the distinct unimodal peak in annual herb density reflects the typical *explosive growth in response to pulsed precipitation* strategy of annual plants in arid zones. Shrub biomass declines continuously with elevation, and its overall magnitude is much lower than that in western regions.

Collectively, unimodal responses represent the dominant pattern across all plant functional groups. Mid-elevations are characterized by optimal hydrothermal coupling, while aridity at low elevations and low temperatures at high elevations impose bidirectional constraints on vegetation performance. Along the longitudinal gradient, vegetation attributes generally show a west-high–east-low declining pattern, with drought stress intensifying progressively from west to east. Annual herbs exhibit the highest sensitivity to moisture gradients.The sensitivity ranking of plant life forms to environmental gradients is as follows: annual herbs > shrubs/semi-shrubs > perennial herbs. Perennial herbs have the widest ecological amplitude and the strongest buffering capacity against environmental gradients.Regional differentiation patterns of desert vegetation are summarized below:

High-value zones: The Ili and Bortala regions (western part) receive precipitation replenishment from westerly airflows, resulting in the most favorable moisture endowment and the highest overall shrub biomass and vegetation cover.
Transition zones: The Tacheng and Altay regions (northern part) support moderate vegetation development, with the most distinct mountain vertical zonation patterns.
Low-value zones: The Eastern Tianshan Mountains (eastern part) are subject to dual constraints of drought and low temperature, with overall sparse vegetation and prominent growth traits of annual herbs.

Annual herbs, characterized by high sensitivity, strong variability, and rapid response to environmental gradients, adopt an opportunistic strategy. Perennial herbs, with the lowest sensitivity and broad ecological amplitude, employ a stress-tolerant strategy. Shrubs and semi-shrubs occupy an intermediate position, adapting to gradient changes through morphological and biomass adjustments. Moisture is the primary driver of east–west differentiation in desert vegetation across northern Xinjiang, while the interplay of thermal conditions and topography shapes vertical and north–south patterns. The Ili Valley constitutes a high-value zone for vegetation development due to its superior moisture endowment, whereas the Eastern Tianshan Mountains form a low-value zone due to extreme aridity.

## Discussion

Most community structural attributes showed unimodal or parabolic trends along elevation across all five subregions, an observational pattern that is descriptively consistent with the expectation of the mid-domain effect (Hypothesis 1). In the Altay subregion, shrub height, coverage, and density all peaked near 800–1000 m. In the Changji-Urumqi subregion, the maximum coverage of all life-forms concentrated in the 700–1000 m range. These elevational unimodal patterns were consistent across subregions, supporting Hypothesis 1 at the descriptive level.

The elevational unimodality observed here may arise from the interplay of geometric range constraints and hydrothermal optimization, but the two mechanisms are confounded in our observational design. At low elevations (200–600 m), high temperatures and intense evapotranspiration create drought stress that limits plant growth, despite the longer growing season. At mid-elevations (700–1100 m), the orographic enhancement of precipitation (400–800 mm in mountainous areas) combined with moderate temperatures creates the optimal hydrothermal window for community structural attribute expression. At high elevations (>1100 m), low temperatures, short growing seasons, and nutrient limitation restrict plant performance.

In the westernmost area on the northern slope of the Tianshan Mountains (e.g., the Ili Valley and the Tekes Valley), the terrain is relatively open. The inflow of warm and humid air currents from the west forms a humid temperate climate in this region.Vegetation structural performance decreased markedly from the relatively humid western subregions (Tacheng-Yili-Bortala) toward the hyper-arid eastern subregions (Changji-Urumqi, Eastern Tianshan), consistent with the west-to-east declining precipitation gradient driven by westerly circulation (Hypothesis 2). This west-to-east aridity gradient is a defining feature of Central Asian deserts, where westerly-derived moisture decreases progressively from the Ili Valley (annual precipitation up to 300–342 mm) to the Eastern Tianshan (Hami, mean annual precipitation = 34 mm). The observed west-to-east decline in community structural attributes is consistent with Hypothesis 2 at the observational level, though longitude is a geographic proxy rather than a direct plot-level drought measurement.

The longitudinal patterns within each subregion consistently reflect this geographic gradient. In the Altay subregion, annual herb cover declined toward 90°E, and annual herb density decreased significantly at the easternmost sampling points. In the Changji–Urumqi subregion, the minimum coverage values of all four life forms were recorded at 90°E. In the Eastern Tianshan—the most arid subregion—subshrub height, subshrub density, perennial herb density, and annual herb density all declined steadily with increasing longitude.

The four plant life-forms exhibited sharply divergent structural patterns along the joint geographic gradients associated with mid-elevation optima and west-to-east aridity, consistent with Hypothesis 3 and reflecting community structural attribute differentiation across the shrubs-herbs continuum.

Shrubs (including small trees such as Haloxylon persicum) and semi-shrubs maintained relatively stable dominance across environmental gradients, a pattern consistent with drought and cold tolerance. In the Altay subregion, shrub and semi-shrub height showed consistent unimodal elevational patterns with peaks at 800–1000 m, and their biomass remained relatively stable across latitudinal and longitudinal gradients. In the hyper-arid Eastern Tianshan, shrub density actually increased continuously with rising elevation, suggesting that woody life-forms can persist under extreme conditions. Perennial herbs favored relatively mesic, mid-elevation habitats and showed stronger sensitivity to water availability than woody life-forms. In the Tacheng subregion, the coverage of perennial herbs increased with rising altitude, but declined significantly at elevations above 1100 m; in the Ili subregion, the height of perennial herbs decreased at elevations above 1200 m. Their observed concentration in mesic mid-elevation habitats is consistent with relatively high soil moisture availability; these species decline rapidly under both high-elevation cold stress and low-elevation aridity. Annual herbs were the most sensitive to water pulses and showed the strongest spatial fluctuations across all gradients. In the Altay subregion, annual herb density was highest at 600 m and decreased sharply above 1000 m; in the Tacheng subregion, annual herb density decreased by 80.4% along the latitudinal gradient; and in the Eastern Tianshan, annual herb density dropped at 45°N after peaking at 44°N. Their opportunistic life-history strategy—germinating, growing, and reproducing rapidly in response to episodic rainfall—is consistent with high responsiveness to water availability, with populations showing the strongest declines in the most arid eastern zones.

These structural differences among life-forms correlate with spatial community assembly in mountain temperate deserts: along the combined geographic gradients, shrubs and semi-shrubs provide the stable structural framework, perennial herbs track the mesic mid-elevation zone, and annual herbs respond dynamically to water availability at the arid margins. This life-form differentiation provides a descriptive framework for understanding the observed community structural attribute patterns across the shrubs-herbs continuum, though the physiological mechanisms underlying these patterns were not directly measured.

Based on the observed spatial patterns, several management and policy implications can be proposed as hypotheses requiring field validation. First, the mid-elevation zone (700–1100 m), which supports the highest community structural values across most subregions, should be prioritized for conservation attention. Protecting this zone may be a cost-effective strategy for maintaining biodiversity and ecosystem services in temperate desert landscapes. Second, the Eastern Tianshan subregion—with the lowest coverage, density, and biomass—warrants prioritized monitoring and possibly stricter grazing controls. However, the specific thresholds and management intensity require dedicated grazing-intensity surveys and restoration trials. Third, the Tacheng-Yili subregion, with relatively high and stable vegetation coverage, may be suitable for scientifically planned rotational grazing, but appropriate stocking rates must be determined through experimentation and socioeconomic assessment.Life-form-specific management is also warranted. The stability of shrubs and semi-shrubs across gradients suggests that woody vegetation should be retained as the structural framework of desert ecosystems, particularly under aridification. Perennial herbs, concentrated in mesic mid-elevation habitats, could serve as indicators of habitat quality and may benefit from targeted water-management measures. The strong responsiveness of annual herbs to episodic rainfall underscores the need for flexible grazing policies that allow for natural regeneration in wet years. Policy frameworks should incorporate these spatial and life-form heterogeneities rather than applying uniform management across the entire region. However, all recommendations remain observational in origin and must be validated through controlled experiments, long-term monitoring, and engagement with local stakeholders before large-scale implementation.

This study examined spatial patterns of community structural attributes (height, coverage, density, aboveground biomass) across five subregions and four plant life-forms along elevational, latitudinal, and longitudinal gradients in the mountain temperate deserts of northern Xinjiang, based on 527 plots surveyed in a single growing season. The main findings are summarized below.

(1) Elevation was the strongest geographic gradient associated with community structural variation. Most attributes showed unimodal or parabolic patterns, with peaks at mid-elevations (700–1100 m) across most subregions. This pattern is consistent with both the mid-domain effect expectation and environmental filtering by hydrothermal gradients, but the present data cannot distinguish between the two mechanisms. Hypothesis 1 is supported by the observational data.
(2) Community structural performance declined from west to east across subregions. The Eastern Tianshan (mean annual precipitation = 34 mm) had the lowest observed values, while the Tacheng-Yili subregion remained relatively stable. A biogeographic threshold occurred near 90°E. Longitude serves as a broad-scale geographic proxy for aridity rather than a direct drought measurement. Hypothesis 2 is partially supported: the cross-subregion pattern is clear, but within-region longitudinal trends are weaker and more variable.
(3) The four life-forms showed divergent structural patterns. Shrubs and semi-shrubs maintained relatively stable dominance across gradients; perennial herbs favored mesic mid-elevation habitats; annual herbs showed the strongest fluctuations in response to water availability. These structural differences are consistent with life-form-specific environmental associations, but physiological mechanisms were not directly measured. Hypothesis 3 is supported by the observational data.

Direct plot-level drought measurements (soil moisture, vapor pressure deficit), tests of spatial autocorrelation, and controlled grazing experiments are priorities for future work. Long-term monitoring across elevation, latitude, and longitude transects is needed to validate these snapshot patterns and detect temporal shifts under climate change.

## Supporting information

S1 Data Dataset. (ZIP)
